# Late Pleistocene climatic changes promoted demographic expansion and population reconnection of a Neotropical savanna-adapted bird, *Neothraupis fasciata* (Aves: Thraupidae)

**DOI:** 10.1371/journal.pone.0212876

**Published:** 2019-03-20

**Authors:** Cássia Alves Lima-Rezende, Amanda Vaz Rocha, Antônio Felipe Couto Júnior, Éder de Souza Martins, Vinicius Vasconcelos, Renato Caparroz

**Affiliations:** 1 Laboratório de Genética e Biodiversidade, Departamento de Genética e Morfologia-IB, Universidade de Brasília, Brasília, Distrito Federal, Brazil; 2 Faculdade UnB Planaltina, Universidade de Brasília, Planaltina, Distrito Federal, Brazil; 3 Embrapa Cerrados, Planaltina, Distrito Federal, Brazil; 4 Laboratório de Solos e Vegetação, Departamento de Ecologia-IB, Universidade de Brasília, Brasília, Distrito Federal, Brazil; Institute of Systematics and Evolution of Animals Polish Academy of Sciences, POLAND

## Abstract

We performed phylogeographic and genetic structure analyses of *Neothraupis fasciata* joined with species distribution modelling to evaluate whether: (1) the distribution of genetic variability shows a pattern expected by the isolation-by-distance model; (2) the influence of the Pleistocene climate changes on species distribution; and (3) climate/climatic stability (hypothesis of climatic stability) as a predictor of population genetic diversity. Based on two molecular datasets (ND2 and FIB-5), the isolation-by-distance hypothesis was not supported. The mitochondrial haplotype network indicated the existence of historically isolated populations at the southern range of the species distribution, and recent population expansion was identified by both neutrality tests and extended Bayesian skyline plot analysis. Thus, the climatic changes during the Pleistocene might have promoted the reconnection of the partially isolated southern populations, which may have persisted in the plateaus during the cycles of savanna contractions. Subsequently, this species (re)colonized northern areas of the species present distribution, following the continuous vegetation on the São Francisco and Central plateaus about 60 kyr, and also reached the Amazonian savannas likely via the central corridor. Thus, our results indicated that the intrinsic relationship between the relief heterogeneity (plateaus and depressions) and the climatic fluctuations, mainly in the Pleistocene, promoted population reconnection and demographic expansion of *N*. *fasciata*.

## Introduction

Tertiary geological events and the Quaternary climatic changes have been considered the key events related to the biodiversity diversification (*e*.*g*. [1–5]). Specifically, the Quaternary climatic cycles had great importance in South American forest and nonforest vegetation (e.g. Savannas) range dynamics [1,3,6–12]. Briefly, it is postulated that during moist and warm periods (interglacial periods), forests expanded their ranges, while during the cold and dry periods (glacial periods) the savannas reached their maximum extension (*see* [9]). Events of both range contraction and expansion during glacial periods have been hypothesized for the "morphoclimatic domain of the Cerrado" [13,14], hereafter referred to as the Cerrado. For instance, during the glacial period the current range of this savanna was replaced by Araucaria forests at its southern edge and by xeric forests at its northern edge, while it was expanded throughout eastern and central Amazonia [14]. According to Werneck *et al*. [3], during the Last Interglacial (LIG, *c*. 120,000 years ago or 120 kyr) the Cerrado expanded, while during the Last Glacial Maximum (LGM, *c*. 21 kyr) the Cerrado reached its smallest range. It is also postulated that during the Pleistocene the forest and nonforest vegetation range dynamics may have led to the establishment of biogeographic corridors currently connecting the isolated savanna blocks in northern Amazonia Forest (Llanos and Amazonian savannas) and Cerrado [1,9,15–20]. The dynamic of range shifts of the Cerrado is complex and there is still no clear pattern related to the diversification of the Cerrado biodiversity that has emerged driven by Pleistocene climatic fluctuations. Considering that the Cerrado is one of the biodiversity hotspots [21–23], understanding the processes that led to the diversification of its biodiversity is also fundamental for its conservation.

The demographic history of the South American species occurring in open vegetation formations was probably influenced by the climatic fluctuations, with expected events of population expansion (*see* leading-edge model [24,25]) following the expansion of suitable habitats and population bottlenecks or extinctions (*see* rear-edge model [24,25]), in response to the retraction of habitat suitability [3,11]. Cycles of population expansion and/or contraction may promote secondary contact among allopatric populations or different episodes of colonization, resulting in different genetic signals. For events of secondary contact, an increase in genetic diversity in the population that received more migrants is expected, since migrants are likely to carry new alleles to the receiver population (*see* [26]). On the other hand, colonization events from a core population lead to a gradual reduction in genetic diversity towards newly colonized areas due to genetic drift operating through repeated founder events or bottlenecks [24–27]. Additionally, when the equilibrium between migration and genetic drift is reached, patterns of genetic diversity may be also explained by the isolation-by-distance model, in which geographically close individuals tend to be genetically more similar than individuals that are further apart [28,29]. Finally, areas that remained climatically stable through the Quaternary climatic changes are expected to exhibit higher genetic diversity than unstable ones, since unstable areas must have experienced more extinction and recolonization events than stable areas [30,31].

Herein, we performed phylogeographic and population genetic analysis of *Neothraupis fasciata* (Lichtenstein, 1823) to evaluate the response of this savanna-adapted bird to environmental changes during the late Quaternary (last 120 kyr). Commonly known as a White-banded Tanager, it is a passerine species from the Thraupidae family, and is the only representative of the genus. This tanager is widely distributed and very common in the undisturbed Cerrado, occurring mainly in cerrado *sensu stricto* and grassy cerrado [32–35]. This species is resident and territorial [36,37], has a low dispersal capacity, with a mean dispersal distance of 200 meters per breeding season [37,38]. A previous study using a set of microsatellite markers found moderate genetic structure in *N*. *fasciata* and the distribution of the genetic variability was not explained by the isolation-by-distance hypothesis, habitat heterogeneity or by the core-periphery effect [39]. Similarly, the intense biome fragmentation by anthropic activity has not contributed significantly to the current pattern of genetic structure found in this species [39]. These findings suggest the influence of historical processes on the distribution of genetic diversity of this tanager.

Based on molecular data sets and ecological niche modeling, we evaluated: (1) the isolation-by-distance hypothesis, with an increase in genetic differentiation among locations being expected due to an increase in geographic distances; (2) the influence of late Quaternary climate changes on the species distribution and demographic history, with events of population expansion being expected in response to the increase in suitable areas and a reduction in genetic diversity following population expansion; and (3) climate/climatic stability (hypothesis of climatic stability) as a predictor of population genetic diversity, with greater genetic diversity in stable areas being expected than in unstable ones.

## Materials and methods

### Species distribution modeling

The species distribution models were estimated compiling occurrence records of *N*. *fasciata* from fieldwork of the Laboratório de Genética e Biodiversidade da Universidade de Brasília, scientific articles, museums, and using the two online databases (Global Biodiversity Information Facility: http://www.gbif.org/; SpeciesLink: http://splink.cria.org.br/). We filtered the 176 unique occurrence points found by excluding redundant records in each pixel on the scale of the bioclimatic variables, resulting in 158 points. A set of 19 bioclimatic variables and elevation for the present were downloaded from WorldClim website in a 2.5 arc-min resolution (http://www.worldclim.org/). The highly correlated variables (|r|≥0.75) were eliminated, and variables with |r|<0.75 were retained using findCorrelation function available on caret (Classification And Regression Training) package [40] in R 3.4.2 [41]. The correlation between the retained bioclimatic variables (Bio2, Bio3, Bio10, Bio13, Bio15, Bio18, and Bio19) ranged from -0.579 (Bio10 ~ Bio18) to 0.531 (Bio3 ~ Bio 15).

The maximum entropy approach implemented in MAXENT 3.4.1 [42] was used to build distribution models [43]. Distribution models were run with 20 replicates subsample, using 20% of the points for test, 10,000 background points, 1,500 maximum iterations and other parameters set as default. Discrimination capacity models were evaluated based on the average values of the area under the curve (AUC), sensitivity, specificity and accuracy. We used a set of seven non-correlated bioclimatic variables to estimate the species distribution under mid-Holocene (6 kyr), LGM, and LIG climate scenarios. Past conditions were based on two general circulation models (CCSM4 and MIROC-ESM) for Holocene and LGM, and the data simulated by Otto-Bliesner *et al*. [44] for the LIG. All bioclimatic datasets were downloaded in a 2.5 arc-min resolution, except for LIG, which was downloaded at 30 arc-sec resolution and exported to a 2.5 arc-min resolution by calculating the mean value for each variable using the aggregate tool in ARCMAP 10.1 (Esri).

To convert habitat suitability into presence/absence binary maps, we used the equal training sensitivity and specificity threshold. For Holocene and LGM we combined the two binary maps obtained with the CCSM4 and MIROC-ESM general circulation models into a single map, in which only areas with predicted occurrence in both projections were highlighted as potential distribution area. Stable areas over the last 120 kyr were delimited based on binary maps and were defined as areas with potential distribution concordant in all projected periods.

To explore the relief, we used the Global Multi-resolution Terrain Elevation Data 2010 (GMTED 2010 - https://earthexplorer.usgs.gov/), which provides a high level of detail in global topographic data 30 arc-sec, suitable for various regional and continental applications [45]. From the mean elevation data (~ 1km in the equator), we derived the slope (percent) and minimum curvature (°.m-1) of Brazil using the System for Automated Geoscientific Analyses (SAGA) software, version 6.2 [46]. We used the elevation, slope and minimum curvature as input grids to run K-mean clustering based on the combined minimum distance [47] and Hill-climbing [48] algorithm, aiming to divide two geomorphological compartments: plateaus and depressions. Considering the habitat preference of *N*. *fasciata*, we evaluated the continuity of the plateaus, based on the categories by Riitters *et al*. [49,50] and the geomorphologic features to delimit the historical population by digital vectorization on a computer screen.

### Genetic sampling

**Fig 1:**
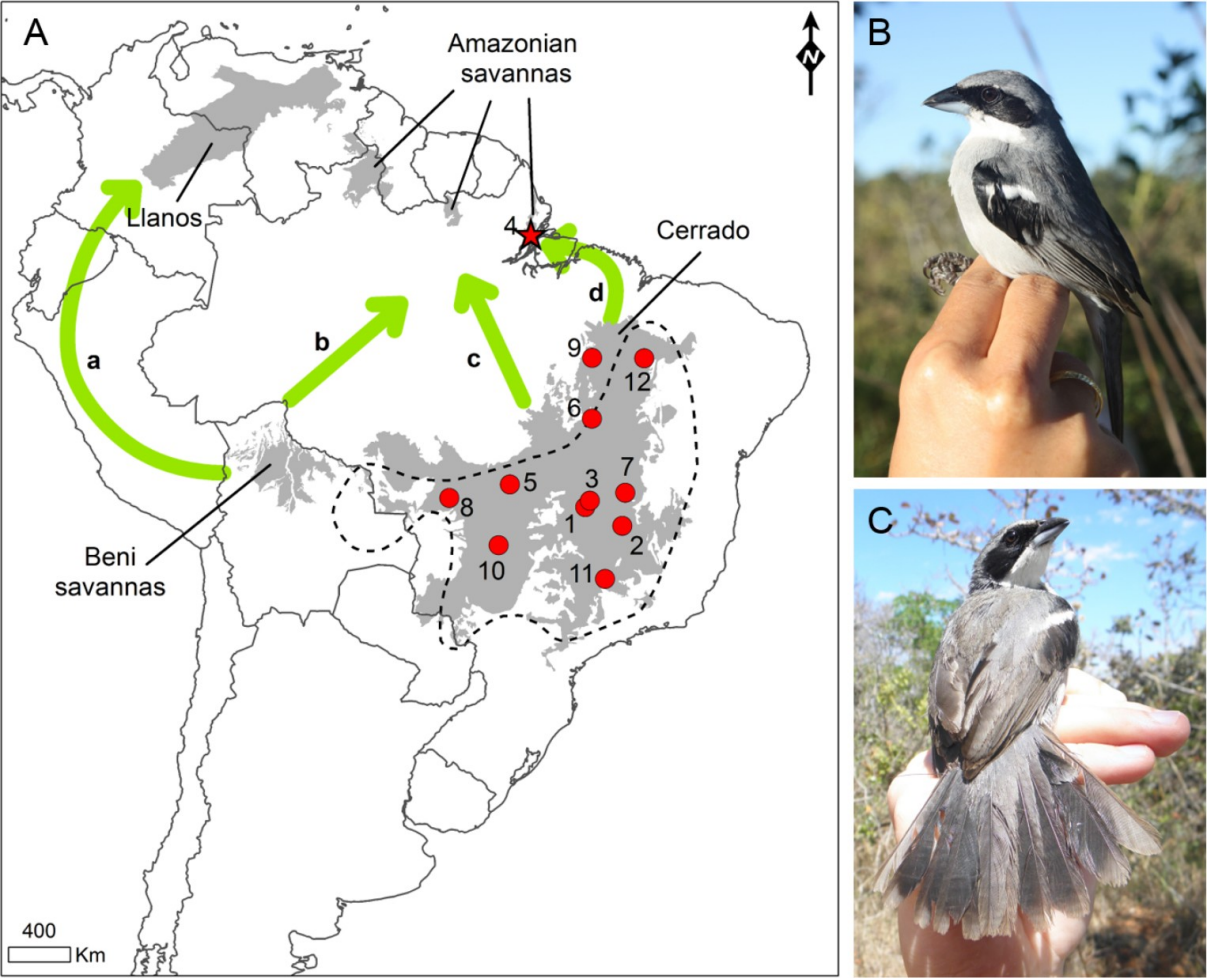
Sample sites of *Neothraupis fasciata* in the Cerrado (red circles) and in the Amazonian savannas of Amapá (red star). (A) The geographic distribution of the species is delimited by a dashed line [51], while the main South American savannas are shown in gray [52]. The green arrows indicate the savanna connections (revised by [1]) (a) along the Andes region [20], (b) following the Madeira River [18], (c) directly across central Amazonia [15] and (d) along the Atlantic coast [15]. Sample sites: 1- Área de Proteção Ambiental das Bacias do Gama e Cabeça de Veado (AGCV); 2- Brasilândia de Minas (BRMI); 3- Estação Ecológica de Águas Emendadas (EEAE); 4- Embrapa Macapá Experimental Farm (MACA); 5- Ouro e Prata Farm at Nova Xavantina (NXAV); 6- Nova Aliança Farm at Ponte Alta do Tocantins (PATO); 7- Parque Nacional Grande Sertão Veredas (PGSV); 8- Parque Nacional Chapada dos Guimarães (PNCG); 9- Parque Nacional da Chapada das Mesas (PNCM); 10- Parque Nacional das Emas (PNEM); 11- Parque Nacional Serra da Canastra (PNSC); and 12- Uruçuí (URUC). (B) Lateral profile of an adult male of *N*. *fasciata* captured in AGCV. (C) Dorsal profile of the adult male captured in AGCV.

Tissue samples were collected from 105 *N*. *fasciata* individuals (Fig 1B and 1C) from 12 locations covering a broad range of the species distribution throughout the Cerrado: Área de Proteção Ambiental das Bacias do Gama e Cabeça de Veado (AGCV), Estação Ecológica de Águas Emendadas (EEAE), Ouro e Prata Farm at Nova Xavantina (NXAV), Nova Aliança Farm at Ponte Alta do Tocantins (PATO), Parque Nacional Grande Sertão Veredas (PGSV), Parque Nacional Chapada dos Guimarães (PNCG), Parque Nacional da Chapada das Mesas (PNCM), Parque Nacional das Emas (PNEM), Parque Nacional Serra da Canastra (PNSC), and in an Amazonian Savanna patch at Embrapa Macapá Experimental Farm (MACA) (Fig 1A and Table 1). In these areas, individuals were attracted by playback and captured using mist-nets. Blood samples (50–100 μL) were taken from each individual by pricking the brachial vein with a sterile needle and collected with a microcapillary tube, then stored in absolute ethanol. All captured birds were ringed with standard ornithological metal rings supplied by Centro Nacional de Pesquisa e Conservação de Aves Silvestres (CEMAVE) and afterwards released at the capture site. Seven ethanol-preserved muscle from individuals sampled at Brasilândia de Minas (BRMI) and Uruçuí (URUC) were kindly provided by Museu Paraense Emílio Goeldi (Table 1). All work was conducted under the approval of the Ethics Committee of the Universidade de Brasília (UnBDoc n° 75111/2013) and research permits were issued by the Brazilian Environmental Agency Instituto Chico Mendes de Conservação (ICMBio) (SISBIO n° 27682–1).

**Table 1:** Summary statistics for NADH dehydrogenase subunit 2 gene (ND2) and beta-fibrinogen gene intron 5 (FIB-5) for *Neothraupis fasciata* from the Cerrado and an Amazonian savanna (MACA). Overall estimate of genetic diversity (S, H, Hd and Nd) was obtained by grouping all individuals as a single population. For sample site names see Fig 1.

Sample site	Label	N	ND2				FIB-5			
			S	H	Hd	Nd (%)	S	H	Hd	Nd (%)
AGCV	1	13	9	8	0.897	0.291	4	5	0.702	0.222
BRMI	2	3	10	2	0.667	0.708	9	4	0.867	0.817
EEAE	3	7	7	6	0.952	0.253	4	5	0.824	0.269
MACA	4	9	1	2	0.500	0.053	3	5	0.712	0.234
NXAV	5	2	3	2	1.000	0.318	2	3	0.833	0.240
PATO	6	14	9	8	0.890	0.185	9	11	0.810	0.540
PGSV	7	22	17	8	0.840	0.656	7	7	0.809	0.579
PNCG	8	8	7	6	0.893	0.216	3	4	0.592	0.195
PNCM	9	5	4	2	0.400	0.170	2	3	0.644	0.224
PNEM	10	9	12	4	0.583	0.283	9	8	0.745	0.513
PNSC	11	9	11	3	0.722	0.478	7	5	0.719	0.492
URUC	12	4	1	2	0.500	0.053	7	5	0.857	0.506
Overall		105	53	36	0.868	0.452	16	23	0.772	0.435

Sample size (N), number of segregating sites (S), number of haplotypes (H), haplotypic diversity (Hd) and nucleotide diversity in percentage (Nd%) are given for each sample site.

Total DNA was extracted from blood samples using proteinase K digestion followed by phenol-chloroform extraction [53], while for museum samples, total DNA was extracted using a PureLink Genomic DNA Kit. We amplified the mitochondrial NADH dehydrogenase subunit 2 gene (ND2) by PCR, using primers LMET (J. Groth *apud* [54]) and H6313 [55] and a fragment of the nuclear beta-fibrinogen gene intron 5 (FIB-5) using the primers FIB5 and FIB6 [56]. The ND2 and FIB introns are markers commonly used in phylogeographic studies of birds and have proven to be informative. PCR reactions were performed in a 10 μl volume containing 30 ng of template DNA, 1X PCR buffer, 0.25 mM of each dNTP, 1 mM of each primer, 1.5 mM MgCl2, and 0.5 unit of Taq polymerase (Invitrogen). Thermocycle conditions were set as: 95°C for 7 min, then 35 cycles at 95°C for 1 min / 52°C for 45 s / 72°C for 1 min, followed by a final extension at 72°C for 10 min. We used the Shrimp Alkaline Phosphatase and Exonuclease I protocol for cleanup PCR products according to the manufacturer's instructions. Sequencing reactions were performed with BigDye Terminator v3.1 Cycle Sequencing Kit according to the manufacturer's instructions and PCR products were sequenced in both directions with an ABI PRISM 3130 DNA genetic analyzer (Applied Biosystems) at the Universidade Católica de Brasília.

We edited the sequences in GENEIOUS 6.0.6 (Geneious Co., Wellington, New Zealand) and coded double peaks in both strands of FIB-5 sequence electropherograms using the IUPAC nucleotide code ambiguous positions. In the FIB-5 sequences, we identified alleles with different sizes (14 bp), generating sequences with mixed traces [57] in heterozygous individuals. To solve this sequencing problem, the readable part of the forward sequence trace in which the pair of allelic sequences properly aligned was concatenated with the complement and reverse of the readable part of the reverse sequence trace. The resulting sequence was aligned with the dataset containing homozygous sequences for the indel and the gap between forward and reverse traces was completed with the IUPAC code for any base “N”. The alignments of consensus sequences of each dataset were done using the Muscle algorithm implemented in GENEIOUS. For FIB-5 we resolved the gametic phase using the algorithm Phase implemented in DnaSP 5.10 [58] with default configurations.

### Molecular analyses

The number of polymorphic sites, number of haplotypes, number of unique haplotypes, haplotype and nucleotide diversities were estimated using DnaSP (Table 1). We estimated genetic diversity indices considering the ND2 and FIB-5 gene fragments separately for each sample site. We constructed haplotype networks for each gene fragment using the median-joining algorithm [59] implemented in POPART 1.7 [60] with all parameters set to default. For FIB-5 network we used the phased dataset inferred in DnaSP.

Genetic differentiation between all sample sites was estimated using Φ-statistics (Φ_ST_) in ARLEQUIN 3.5.1.3 [61]. Calculations of Φ_ST_ were done using Tamura-Nei model for ND2 alignment and Jukes-Cantor model for FIB-5 alignment. The best-fit model of evolution of each dataset was selected according to the Bayesian Inference Criterion in JMODELTEST 2.1.10 [62]. We used a total of 50,000 permutations to assess the statistical significance of each pairwise comparison. Genetic groups across the landscape were identified employing a Bayesian approach. The Bayesian Analysis of Population Structure (BAPS) was performed at the population level (spatial clustering of groups), considering each locality as a population and using their respective geographic coordinates in BAPS 6.0 program [63]. To determine the optimal number of genetic groups, BAPS analysis was performed for each marker with the maximum number of groups (*K*) set to 12, and 20 replicates for each *K*-value.

To verify whether the genetic differentiation among each pair of individuals sampled in the Cerrado is associated with the geographic distance, we performed a Mantel test. The Euclidean geographic distances were obtained using an on-line distance calculator from the Instituto Brasileiro de Pesquisas Espaciais (http://www.dpi.inpe.br/calcula/). We conducted the test using the corrected genetic distance (PhiST/(1-PhiST)) and the logarithm of the geographic distance in IBDWS 3.23 [64], considering each locus separately and 30,000 randomizations. We also evaluated the influence of geographic gradients on genetic diversity performing a regression analysis of genetic diversity indices (percentage of nucleotide diversity and haplotype diversity) and geographic variables of all sample locations (latitude and longitude, expressed as decimal degrees). All regressions were done using Spearman’s rank-order correlation coefficient in PAST 3.11 [65].

To evaluate whether climatic stability influenced the genetic diversity, we investigated whether the genetic diversity of Cerrado sample locations was higher in the Quaternary stable areas compared to unstable areas. We classified all locations as belonging to stable or unstable areas based on the stability map. Comparisons were done for each locus using the non-parametric Kruskal-Wallis test in PAST, in which the stability was considered the explanatory variable and the genetic indices (percentage of nucleotide diversity or haplotype diversity) the response variables.

We performed Tajima´s D [66] and Fu´s Fs [67] neutrality tests and estimated the R2 statistic [68] to infer past population expansion considering the structure found by BAPS. All tests were performed in DnaSP using 10,000 coalescent simulations and a significance level of 0.05. We also inferred the dynamic of population size as a function of time performing a coalescent extended Bayesian skyline plot (EBSP) analysis implemented in BEAST 1.8.4 [69]. We used the TN93+I substitution model for ND2 and HKY+I model for the FIB-5. Analyses were done implementing the lognormal relaxed clock model, since the null hypothesis of equal evolutionary rate throughout the tree was rejected for both markers. We set a mutation rate of 9.0x10^-9^ per base pair per year for ND2 [70] and 3.6x10^-9^ per base pair per year for FIB-5 [71]. We used a total of 400 million runs and saved parameters every 40 thousand runs and looked at the posterior effective sample size (ESS) in TRACER 1.6 [72] using a threshold of 200. Finally, we plotted the median of population size in log scale through time and displayed the 95% high posterior density intervals using R.

We evaluated the genetic differentiation (Φ_ST_) and the mitochondrial haplotype sharing between pairs of populations to evaluate whether the proposed biogeographic corridors served as dispersal routes for *N*. *fasciata*, considering: 1) MACA and PNCG—as an indicator of historical connection throughout the Andes region and/or Madeira River; 2) MACA and PATO, AGCV, or EEAE as an indicator of historical connections across central Amazonia; and 3) MACA and URUC or PNCM as an indicator of historical connection along the Atlantic coast. Genetic differentiation was estimated using *F*-statistics as previously described.

## Results

### Species distribution models

**Fig 2:**
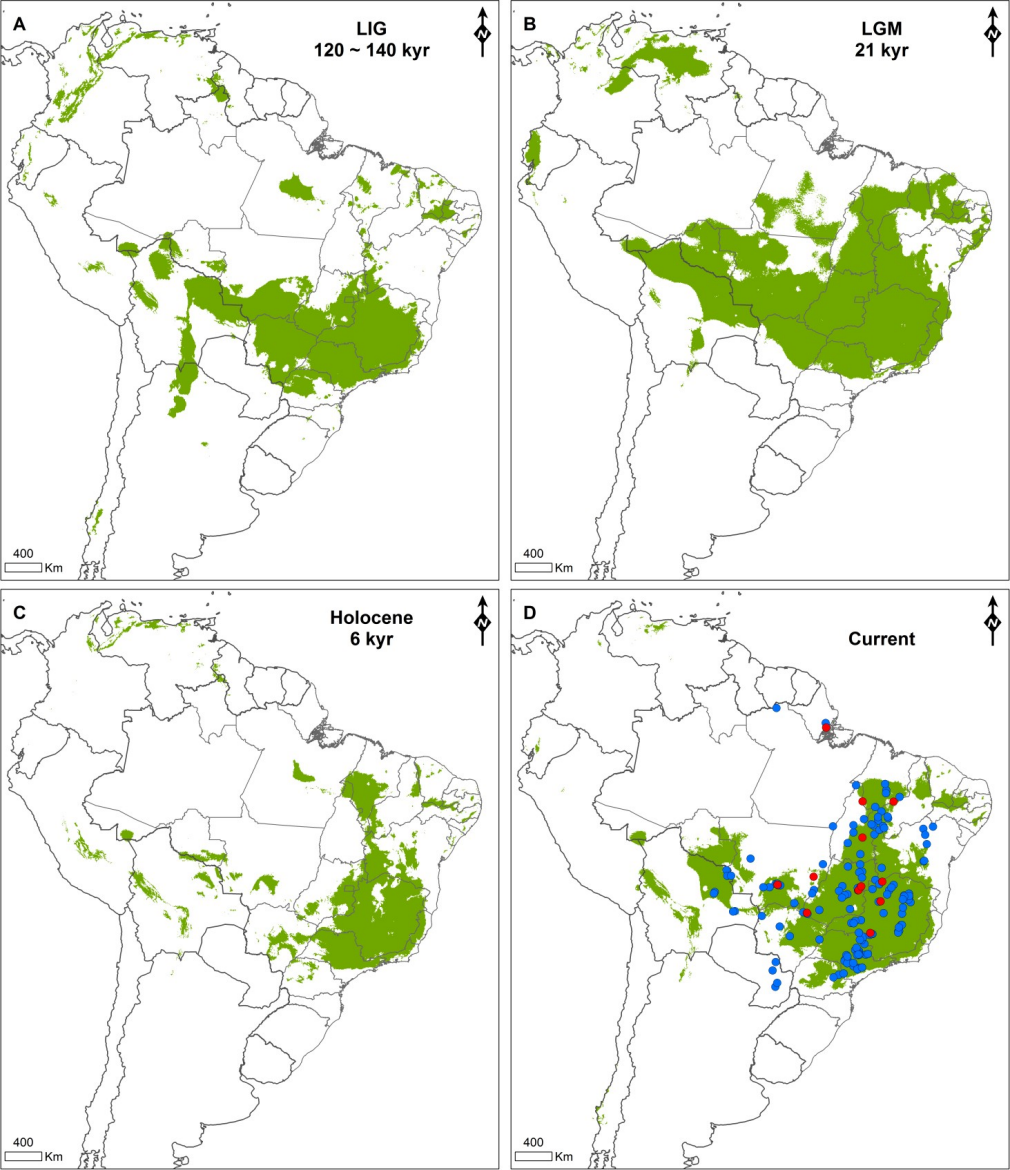
**Species distribution models of *Neothraupis fasciata* for different time projections: (A) Last Interglacial; (B) Last Glacial Maximum; (C) Holocene; and (D) present.** Habitat suitability (green areas) was obtained by applying the equal training sensitivity and specificity threshold. Blue and red dots in D represent occurrence points used in species distribution modelling, while red dots also represent sample sites used in genetic analyses.

A total of 158 occurrence records of *N*. *fasciata* were used to construct the species distribution models (Fig 2D and S1 Table), with most of these records being distributed in Brazilian territory and few of them in Paraguay, Suriname, and Bolivia. The climatic niche model for the present showed a high predictive power, based on the high average AUC value (0.906, S2 Table).

Based on paleoclimatic models, suitable areas during the LIG were more fragmented and restricted to the southern part of the distribution, compared with the projection for the present time (Fig 2). The LGM was characterized by the expansion of suitable areas, mainly in the central and northern Cerrado distribution compared to the LIG. The Holocene seems to be the less favorable climatic period for the species, characterized by a remarkable reduction of suitable areas compared to the LGM. Suitable area patches concordant with the central Amazonia belt of low precipitation were observed during the LIG and Holocene, while small suitable areas concordant with the Andean slopes were found only during the LIG.

**Fig 3:**
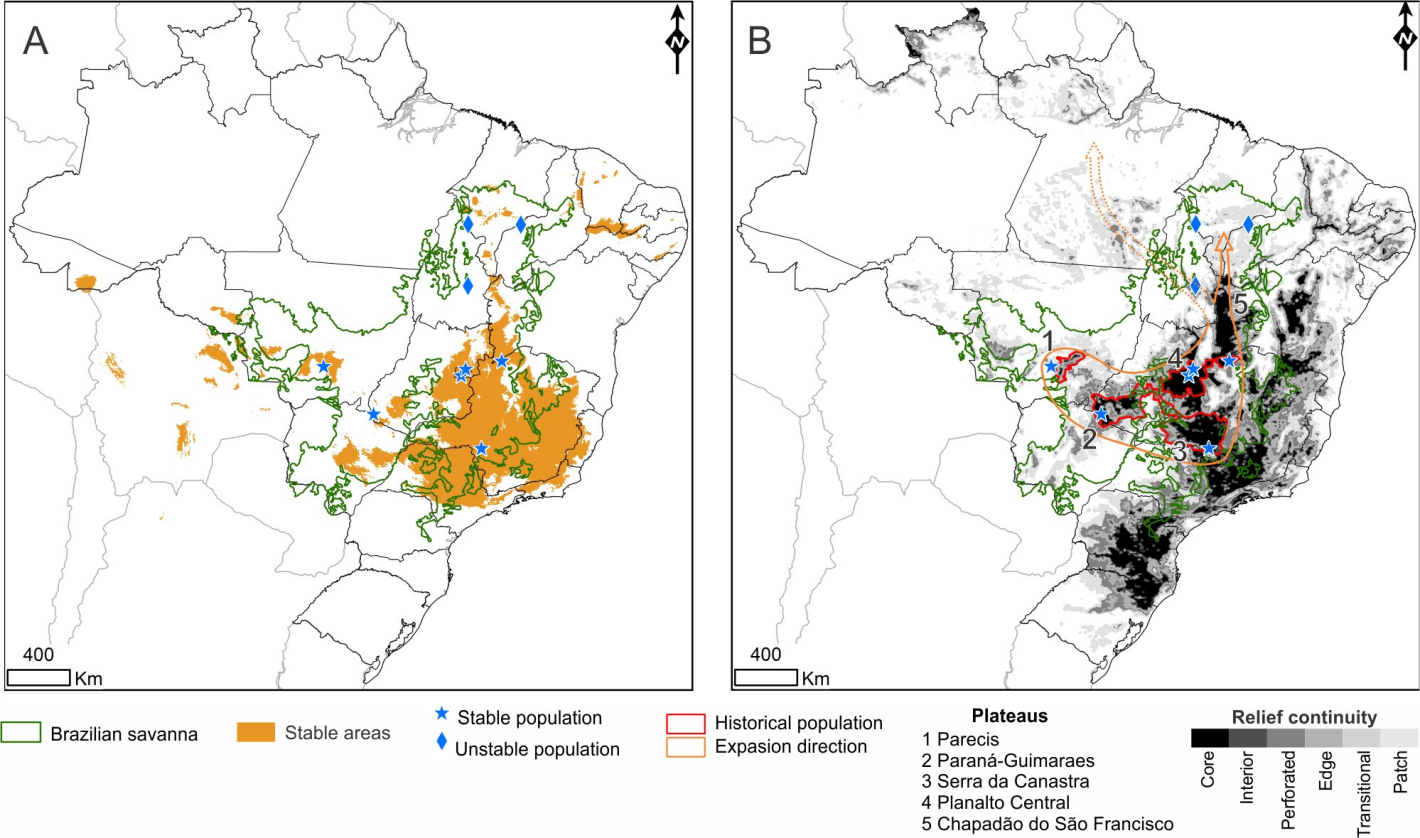
Areas of historical climatic stability, relief continuity and population expansion hypothesis for *Neothraupis fasciata*. (A) Stable areas (orange) correspond to areas with climatic suitability overlap between the Last Interglacial, Last Interglacial Maximum, Holocene and present time projections. Sample sites were categorized as belonging to stable (blue stars) or unstable areas (blue diamonds). (B) Relief continuity (gray and black areas), historical population structure (delimited in red) and recent population expansion in *Neothraupis fasciata* (delimited in orange). The arrows indicate the routes of demographic expansion (about 60 kyr) observed for *Neothraupis fasciata*.

The distribution of the stable areas over the last 120 kyr is roughly concordant with some Brazilian plateaus: Parecis, Paraná-Guimarães, Serra da Canastra, Chapadão do São Francisco and Central (Fig 3B). All of these plateaus have characterized the landscape since the Paleogene (43–20 million years ago—myr) [73,74]. In the last 20 myr, these plateaus have been dissected and denuded as a result of temperature and precipitation increases [74,75], forming depression compartments between the relict relief. The duality between depressions and plateaus generates discontinuous or fragmented compartments. In Central Brazil, the relict relief with the greatest territorial extension is identified as *Core* (Fig 3). Depressions and valleys are located between *Core* compartments where *Interior* and *Edge* classes can be identified (Fig 3B). Currently, patches are observed in the north of the Brazilian Cerrado where the plateaus are less frequent in this region. Considering the distribution of the stable areas, the sampling sites AGCV, EEAE, PGSV, PNEM, PNCG, and PNSC were assigned to stable areas, while URUC, PATO, and PNCM corresponded to areas without climatic suitability (or unstable areas) in at least one of the projections (Fig 3A).

### Molecular data

We obtained ND2 (942 bp) and FIB-5 (487 bp) sequences for all 105 *N*. *fasciata* samples, observing 36 haplotypes for ND2 and 23 for FIB-5 (Table 1). Sequences were deposited in GenBank under accession numbers MH277700—MH277804 (ND2) and MH277805—MH277909 (FIB-5) (S3 Table). Haplotype diversity was lower for the nuclear dataset (0.772) compared to the mitochondrial (0.868) dataset, while nucleotide diversity was similar for both markers (ND2: 0.46%; FIB-5: 0.44%) (Table 1).

**Fig 4:**
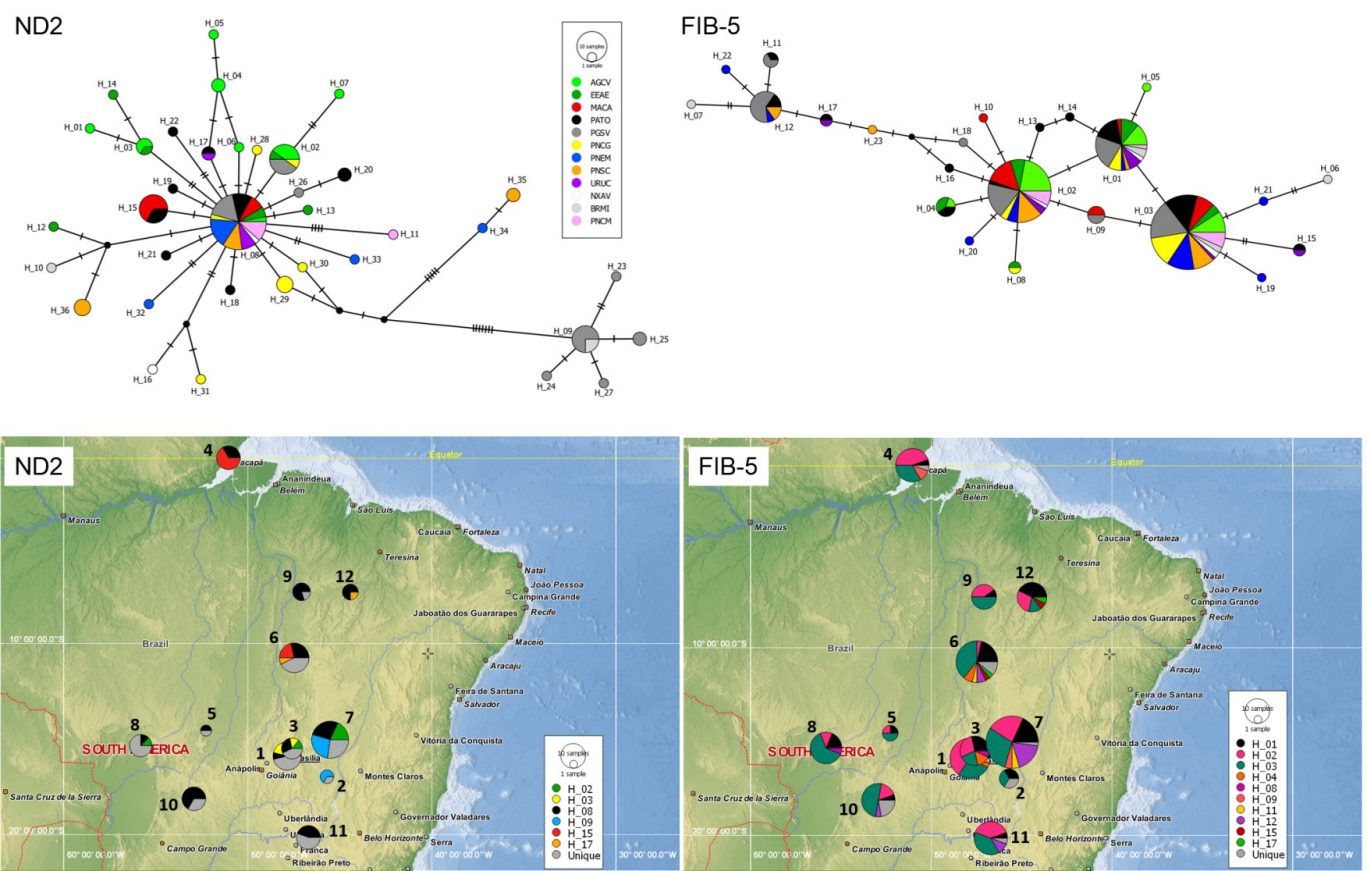
Median joining networks and haplotype distribution maps obtained using NADH dehydrogenase subunit 2 gene (ND2) and beta-fibrinogen gene intron 5 (FIB-5) datasets. In the networks, each haplotype is represented by a numbered and colored circle: colors correspond to the sample sites, and circle size and pie charts are proportional to the number of individuals (see Table 1 and Fig 1 to interpret). Mutational steps are represented by hatch marks and missing haplotypes are indicated by small black circles. In the haplotype maps, colors correspond to haplotypes in the sample site, and the size of circle and pie charts are proportional to the number of individuals (see Table 1 and Fig 1 to interpret). Non-shared haplotypes (unique) are shown in gray.

The ND2 haplotype network was represented by one broadly distributed and common haplotype connected by a few mutational steps with several less frequent haplotypes in a star-like pattern (Fig 4). This pattern can be interpreted as a signal of recent population expansion. Moreover, the ND2 network also showed a haplotype group composed exclusively of individuals sampled in PGSV and BRMI, and a second group comprised of individuals sampled in PNSC and PNEM. Both haplotype groups were separated from the common haplotype by several mutational steps. The FIB-5 haplotype network showed three of the most common haplotypes connected by a maximum of three mutational steps from each other and widely distributed across sample locations (Fig 4). The genetic structure obtained by BAPS analysis was similar for both markers and showed support for only one genetic group.

The results of Mantel tests for both ND2 (r = 0.0059, Z = 53.8917, p = 0.5218) and FIB-5 (r = -0.069, Z = 3.7704, p = 0.662) indicated that the geographic distances do not correlate with the genetic differentiation among sample locations. The analyses of the influence of geographic gradients in genetic diversity using the ND2 indicated a negative correlation between nucleotide diversity and latitude (rs = -0.728, p = 0.007), characterized by a south-to-north trend of declining genetic diversity (Table 2). For FIB-5 there is an east-to-west trend of declining nucleotide diversity (rs = 0.580, p = 0.048) (Table 2). There is a significant difference between ND2 nucleotide diversity sample medians of stable and unstable areas, with greater values in stable areas compared to unstable ones (χ2 = 5.4, p = 0.020) (Table 3). For the remaining comparisons, the differences between stable and unstable areas were not statistically significant (Table 3).

**Table 2:** Calculation of Spearman rank order correlation coefficient (rs) using genetic diversity indices of NADH dehydrogenase subunit 2 gene (ND2) and beta-fibrinogen gene intron 5 (FIB-5) and geographic gradients for *Neothraupis fasciata*.

Marker	Genetic variable	Geographic variable	rs	*p*
ND2	nucleotide diversity	latitude	-0.728	0.007*
	haplotype diversity	latitude	-0.322	0.307
	nucleotide diversity	longitude	0.154	0.632
	haplotype diversity	longitude	-0.371	0.235
FIB-5	nucleotide diversity	latitude	-0.238	0.457
	haplotype diversity	latitude	-0.067	0.829
	nucleotide diversity	longitude	0.580	0.048*
	haplotype diversity	longitude	0.468	0.124

Significant p-values are marked with asterisk (*).

**Table 3:** Kruskal-Wallis tests (H(χ2)) for comparison of genetic diversity vs. habitat stability. Medians of genetic diversity indices of NADH dehydrogenase subunit 2 gene (ND2) and beta-fibrinogen gene intron 5 (FIB-5) are given for stable and unstable areas.

Marker	Genetic indices	Stable	Unstable	H(χ2)	*P*
ND2	nucleotide diversity	0.287	0.170	5.4	0.020*
	haplotype diversity	0.867	0.500	2.4	0.121
FIB-5	nucleotide diversity	0.381	0.506	0.267	0.601
	haplotype diversity	0.732	0.810	0.6	0.439

Significant p-values are marked with asterisk (*).

**Fig 5:**
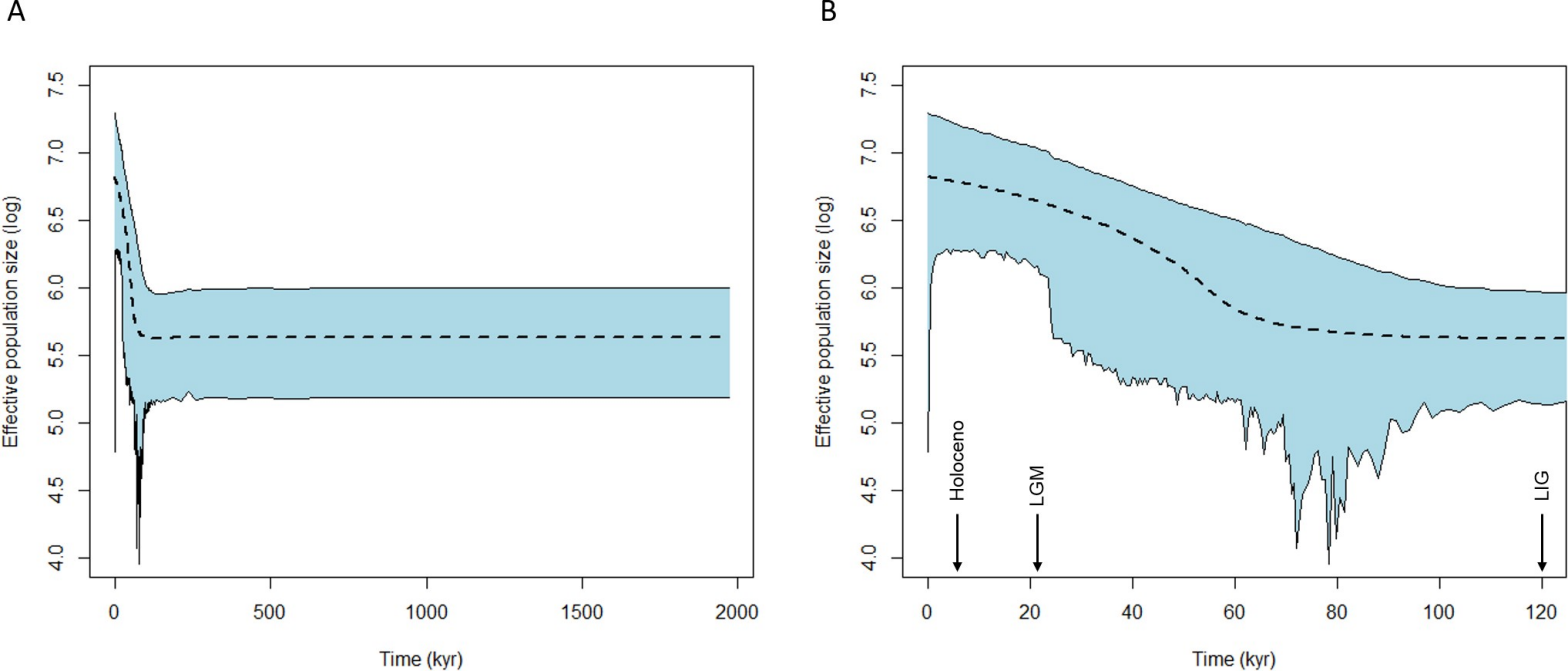
Extended Bayesian skyline plot representing the changes in *Neothraupis fasciata* effective population size (log transformed) over time (in thousand years ago—kyr). (A) the historical demographic trend for the last 2 myr; (B) a detail of the historical demographic trend from the Last Interglacial (~120 kyr) until now. The dashed line represents the median estimate of the effective population size, with the 95% high posterior density interval shown in blue.

Based on the genetic structure found, neutrality tests were carried out considering all sample sites as a single genetic population. Deviations from neutrality were found in Tajima's D (D = -2.9729; p = 0.009), Fu's Fs (Fs = -20.1994; p = 0.000), and R2 (R2 = 0.0374; p = 0.011) tests for the ND2, suggesting recent population expansion. For FIB-5, deviation from neutrality was found for Fu's Fs (Fs = -10.226; p = 0.005), but not for Tajima's D (D = -0.7600; p = 0.235) and R2 (R2 = 0.0636; p = 0.317) tests. EBSP analysis considering both markers together showed a signal of population expansion starting about 60 kyr (Fig 5).

## Discussion

In the present study, we assessed the levels of genetic diversity and structure in *Neothraupis fasciata* based on samples from a wide geographic area in the Cerrado. Our data support a single population (supported by BAPS results) with a weak genetic structure (global Φ_ST_), in which southernmost locations showed more genetic variability than those sampled in the northern Cerrado. Considering that areas of greater genetic diversity are commonly associated with the origin center of a species (*e*.*g*. [25,27,76]), it is plausible to assume that the origin center of the *N*. *fasciata* lineage is in the southern part of the Cerrado. The southern Cerrado encompasses some topographical relief core areas, such as Parecis, Paraná-Guimarães and Serra da Canastra plateaus, which are, at least in part, concordant with some predicted stable areas (see Fig 3). These historically stable areas may have played an important role in the speciation process and also served as refugia favoring higher levels of genetic diversity for this species. Additionally, according to the phylogeny of the Thraupidae family [77], the distribution range of the *N*. *fasciata* sister species (*Lophospingus pusillus*, *Lophospingus griseocristatus*, *Gubernatrix cristata*, and *Diuca diuca*) is restricted to southern South America [78].

The observed genetic structure in *N*. *fasciata* does not seem to be related to the isolation-by-distance hypothesis. This is an unexpected result, given the low dispersal from the birth area reported for this species (around 300 meters; [37]). However, a previous study of the genetic structure of this tanager using microsatellite markers also found no effect of geographic distance under the genetic differentiation among these same sampled locations [39]. The isolation-by-distance pattern occurs when the migration-drift equilibrium is reached, but it can take a relatively long time [79]. Therefore, the demographic history of a species should be considered, since the isolation-by-distance pattern is most commonly found in older populations and may be absent in populations established in a more recent time [80]. Considering that our results suggest a recent population expansion in *N*. *fasciata*, it seems reasonable to assume that the mutation-drift equilibrium has not been reached yet, and this may be masking the effect of the low dispersion capacity of this species in the spatial distribution of its genetic diversity.

The Cerrado landscape is characterized by large blocks of plateaus (Fig 3) separated from each other by peripheral depressions, probably formed at least 2–3 million years ago [13]. Our ecological niche models associated with the topographical information showed that the major stable areas were associated with different plateau areas, akin to previous studies that found stable areas along central Brazilian plateaus [3,17,19,81–83]. Although Ab’Saber [81] suggested the existence of a large and continuous stable area along the plateaus in the southern Cerrado during the Quaternary, this hypothesis was not supported by our findings (Fig 3) and by other studies that found small and isolated stable areas along the southern Cerrado [3,82,83], plausibly representing isolated species refugia.

Our paleodistribution models also showed an increase in suitable area and connection from LIG to LGM, and a decrease and fragmentation from the LGM to the Holocene, followed by an expansion from the Holocene to the present distribution of *N*. *fasciata*. The shifts in the distribution of habitat suitability for *N*. *fasciata* presumably accompanied the shifts in the distribution of the Cerrado itself, since this species is strictly associated with cerrado *sensu stricto* and grassy cerrado [32–35]. In this sense, our results are consistent with the predicted expansion of South American open biomes during cold and dry periods, and retraction during warmer periods (*e*.*g*. [1,7,8,10,11,84]).

According to our results, *N*. *fasciata* underwent a recent population expansion event starting about 60 kyr (neutrality tests and Figs 4 and 5) likely favored by the increasing in area and connection of suitable habitats between LIG and LGM. Although the increase in connection of suitable areas was detected in the projection for 21 kyr, it is possible that this change started earlier. The period from 75 to 12 kyr (marine isotope stages 4–2) was marked by climatic changes reflecting a decrease of the average temperature, characterized by minor glacial cycles [85,86], which may have contributed to the increase in the connection of suitable habitats for *N*. *fasciata*. Although our results indicated an event of population expansion, studies encompassing open-vegetation vertebrates of South America showed different trends for the LGM, with events reported of decrease (mammals and birds), expansion (mammals), or even constant effective population size (mammals and lizards) (see [4]).

We found greater genetic diversity in those individuals from the southern sample sites: (I) PNCG, (II) PNSC + PNEM, and (III) PGSV + BRMI, and a low number sharing haplotypes with each other (Fig 4). These results suggest that these populations were partially isolated before the expansion event. Events of population expansion may result in a gradual reduction in genetic diversity toward newly colonized areas due to successive founder effects (leading-edge expansion model) and the increase in genetic diversity in populations that received migrants [26,27]. Thus, our results indicate that the process of population expansion promoted the reestablishment of gene flow in the southern part of the species distribution, reconnecting genetic groups historically isolated in different plateaus (Fig 3). Additionally, the gradual reduction in genetic diversity following a south-to-north trend indicates that the species may have survived in isolated refugia in the southern part of its distribution during less favorable periods with subsequent colonization events towards the northern Cerrado. This process was likely favored by the continuity of vegetation formed in the core Central plateau and a possible extension of the cerrado sensu stricto-like vegetation toward northern residual relief (see transitional and patch areas in Fig 3B) in the interplateau depressions promoted by the decrease in temperature between LIG and LGM. Plateaus have deep and well-drained soils that are favorable to the establishment of the cerrado *sensu stricto* vegetation, while interplateau depressions have younger soils and, consequently, a more heterogeneous landscape than the plateaus (see Silva 1995 for a review). As in the plateaus, intraplateau depressions are composed of planning surfaces covered predominantly by Oxisols, which favor the establishment of cerrado sensu stricto-like vegetation in response to climatic changes. In this regard, the interplateau depressions are possibly more prone to vegetation range shifts due to climatic changes than the plateaus, as denoted by our paleomodelling results and the literature that have associated the Cerrado stable areas with plateaus and residual relief [3,17,19,81–83].

We observed higher genetic diversity and a signal of historical differentiation between populations located in different stable areas. Thus, the mitochondrial results reinforce the hypothesis that high levels of divergence and population structure are expected across refugia [31] and the genetic diversity is expected to be higher in stable areas compared to unstable areas [19,30,31,83]. In addition, we stress that *N*. *fasciata* sample locations categorized as unstable are in the northern part of the distribution, and the effects of range expansion may act as a confounding factor. Therefore, additional sampling mainly in unstable areas is necessary to disentangle the effects of population range expansion and historical stability.

Connections among the South American savannas are postulated to have occurred through biotic corridors: along the Andes region [20]; right across central Amazonia, following a belt of low precipitation [15]; and along the Atlantic coast [84] (see [1] for a review). Additionally, a fourth corridor crossing Amazonia from the southwest to the northeast following the Madeira River was postulated as an alternative dispersal route [18]. Our results showed that MACA and PATO hold the lowest level of genetic differentiation (S4 Table) and are the only ones to share haplotypes, suggesting that *N*. *fasciata* dispersal may have occurred mainly through central Amazonia corridor, besides we were not able to dismiss the use of the other hypothesized corridors. Additionally, the niche models indicated a region of habitat suitability concordant with the low precipitation belt during the LIG. In accordance, Bueno *et al*. [17] and Buzatti *et al*. [19] reported the existence of suitable climatic conditions to establish different Cerrado tree species throughout central Amazonia, reinforcing the existence of the central Amazonian corridor. However, our results are not in accordance with the literature that suggests the corridors along the Atlantic coast [1] or the Madeira River [18] as the main dispersal routes used by *N*. *fasciata*. The pattern observed in our results and the growing literature about the importance of the central Amazonian corridor (e.g. [17,19]) highlight the need for more studies to understand the importance of the hypothesized Quaternary savanna corridors to the South American avifauna dispersal.

## Conclusion

Genetic groups of *N*. *fasciata* located in the southern part of the species distribution (likely its center of origin) might have persisted in partially isolated populations restricted to the plateaus during the cycles of savanna contractions. Subsequent events of savanna expansion promoted the reestablishment of gene flow among these isolated populations and the increase in the distribution range through the (re)colonization of the northern portion of the species distribution. Thus, the intrinsic relationship between the relief heterogeneity (plateaus and depressions) and the climatic fluctuations, mainly in the Pleistocene, promoted population reconnection and demographic expansion of *N*. *fasciata*, reinforcing previous studies (*e*.*g*. [82]) that suggest that this relation of cause and effect (the interaction between climate and relief defining vegetation dynamic) may have been decisive in the diversification of the Cerrado biodiversity.

## Supporting information

S1 Table *Neothraupis fasciata* occurrence points. (DOCX)

S2 Table Summary of performance statistics of *Neothraupis fasciata* MAXENT models. The average Area Under the ROC curve (AUC) for the replicate runs (standard deviations in brackets) is given for each model. The average test omission rate (OR) is also given for each model.
(DOCX)

S3 Table Genbank accession numbers and haplotypes for NADH dehydrogenase subunit 2 gene (ND2) and beta-fibrinogen gene intron 5 (FIB-5) datasets from *Neothraupis fasciata* individuals sampled along 12 different sample sites. See Fig 4 for haplotype number (Hap) interpretation. Área de Proteção Ambiental das Bacias do Gama e Cabeça de Veado (AGCV); Brasilândia de Minas (BRMI); Parque Nacional da Chapada das Mesas (PNCM); Estação Ecológica de Águas Emendadas (EEAE); Embrapa Macapá Experimental Farm (MACA); Ouro e Prata Farm at Nova Xavantina (NXAV); Nova Aliança Farm at Ponte Alta do Tocantins (PATO); Parque Nacional Grande Sertão Veredas (PGSV); Parque Nacional Chapada dos Guimarães (PNCG); Parque Nacional das Emas (PNEM); Parque Nacional Serra da Canastra (PNSC); and Uruçuí (URUC).
(DOCX)

S4 Table Pairwise Φ_ST_ values for NADH dehydrogenase subunit 2 gene (bellow diagonal) and beta-fibrinogen gene intron 5 (above diagonal) datasets for *Neothraupis fasciata* sample sites (see Fig 1 for abbreviations). Significant p-values after False Discovery Rate correction are marked with one asterisk (*).
(DOCX)

S1 Fig *Neothraupis fasciata* occurrence points and predicted present distribution based the set of seven non-collinear variables (Bio2, Bio3, Bio10, Bio13, Bio15, Bio18, and Bio19). Warmer colors represent areas of higher habitat suitability.
(DOCX)

S2 Fig ***Neothraupis fasciata* occurrence points and predicted Holocene distribution using (A) MIROC and (B) CCSM4 General Circulation Models.** Warmer colors represent areas of higher habitat suitability.
(DOCX)

S3 Fig ***Neothraupis fasciata* occurrence records and predicted Last Glacial Maximum distribution using (A) MIROC and (B) CCSM4 General Circulation Models.** Warmer colors represent areas of higher habitat suitability.
(DOCX)

S4 Fig *Neothraupis fasciata* occurrence points and predicted Last Interglacial distribution. Warmer colors represent areas of higher habitat suitability.
(DOCX)

## References

[1] Silva JMC. Biogeographic analysis of the South American Cerrado avifauna. Steenstrupia. 1995;21: 49–67.

[2] Colli GR. As origens e a diversificação da herpetofauna do Cerrado In: Scariot A, Sousa-Silva JC, Felfili JM, editors. Cerrado: Ecologia, Biodiversidade e Conservação. Brasília, DF: Ministério do Meio Ambiente; 2005 pp. 247–264.

[3] Werneck FP, Nogueira C, Colli GR, Sites JW Jr, Costa GC. Climatic stability in the Brazilian Cerrado: Implications for biogeographical connections of South American savannas, species richness and conservation in a biodiversity hotspot. J Biogeogr. 2012;39: 1695–1706.

[4] Turchetto-Zollet AC, Pinheiro F, Salgueiro F, Palma-Silva C. Phylogeographical patterns shed light on evolutionary process in South America. Mol Ecol. 2013;22: 1193–1213. 10.1111/mec.12164 23279129

[5] Bennett KD. Continuing the debate on the role of Quaternary environmental change for macroevolution. Philos Trans R Soc B. 2004;359: 295–303. 10.1098/rstb.2003.1395 15101585 PMC1693323

[6] Haffer J. Speciation in Amazonian forest birds. Science. 1969;165: 131–137. 10.1126/science.165.3889.131 17834730

[7] Bigarella JJ, Andrande D, Riehs PJ. Considerações a respeito das mudanças paleoambientais na distribuição de algumas espécies vegetais e animais no Brasil. Simpósio Int sobre o Quartenário. 1975;47: 411–464.

[8] Burnham RJ, Graham A. The history of Neotropical vegetation: New developments and status. Ann Missouri Bot Gard. 1999;86: 546–589.

[9] Silva JMC, Bates JM. Biogeographic patterns and conservation in the South American Cerrado: A tropical savanna hotspot. Bioscience. 2002;52: 225–233.

[10] Mayle FE, Beerling DJ. Late Quaternary changes in Amazonian ecosystems and their implications for global carbon cycling. Palaeogeogr Palaeoclimatol Palaeoecol. 2004;214: 11–25.

[11] Carnaval AC, Moritz C. Historical climate modeling predicts patterns of current biodiversity in the Brazilian Atlantic forest. J Biogeogr. 2008;35: 1187–1201.

[12] Leite YLR, Costa LP, Lossa AC, Rocha RG, Batalha-Filho H, Bastos AC, et al Neotropical forest expansion during the last glacial period challenges refuge hypothesis. Proc Natl Acad Sci. 2016;113: 1008–1013. 10.1073/pnas.1513062113 26755597 PMC4743791

[13] Ab’Sáber A. Os domínios de natureza no Brasil: Potencialidades paisagísticas 4a. São Paulo, SP: Ateliê Editorial; 2003.

[14] Ab’Sáber A. Os domínios morfoclimáticos da América do Sul: Primeira aproximação. Geomorfologia. 1977;52: 1–21.

[15] Haffer J. Notas zoogeográficas sobre las avifaunas de las regiones no forestadas de Sudamérica noroccidental. El Hornero. 1967;10: 315–333.

[16] Werneck FP. The diversification of eastern South American open vegetation biomes: Historical biogeography and perspectives. Quat Sci Rev. 2011;30: 1630–1648.

[17] Bueno ML, Pennington RT, Dexter KG, Kamino LHY, Pontara V, Neves DM, et al Effects of Quaternary climatic fluctuations on the distribution of Neotropical savanna tree species. Ecography (Cop). 2016;39: 1–12.

[18] Ribeiro V, Werneck FP, Machado RB. Distribution dynamics of South American savanna birds in response to Quaternary climate change. Austral Ecol. 2016;41: 768–777.

[19] Buzatti RSO, Pfeilsticker TR, Magalhães RF, Bueno ML, Lemos-Filho JP, Lovato MB. Genetic and historical colonization analyses of an endemic savanna tree, *Qualea grandiflora*, reveal ancient connections between Amazonian Savannas and Cerrado. Front Plant Sci. 2018;9: 1–16. 10.3389/fpls.2018.00001 30065733 PMC6056688

[20] Webb SD. A history of savanna vertebrates in the new world. Part II: South America and the Great Interchange. Annu Rev Ecol Syst. 1978;9: 393–426.

[21] Myers N, Mittermeier RA, Mittermeier CG, Fonseca GA, Kent J. Biodiversity hotspots for conservation priorities. Nature. 2000;403: 853–858. 10.1038/35002501 10706275

[22] Klink CA, Machado RB. A conservação do Cerrado brasileiro. Megadiversidade. 2005;1: 147–155.

[23] Marchese C. Biodiversity hotspots: A shortcut for a more complicated concept. Glob Ecol Conserv. 2015;3: 297–309.

[24] Hampe A, Petit RJ. Conserving biodiversity under climate change: The rear edge matters. Ecol Lett. 2005;8: 461–467. 10.1111/j.1461-0248.2005.00739.x 21352449

[25] Hewitt GM. The genetic legacy of the Quaternary ice ages. Nature. 2000;405: 907–913. 10.1038/35016000 10879524

[26] Gillespie RG, Roderick GK. Geology and climate drive diversification. Nature. 2014;509: 297–298. 10.1038/509297a 24828187

[27] Merilä J, Bjorklund M, Baker AJ. Historical demography and present day population structure of the greenfinch, *Carduelis chloris*—An analysis of mtDNA control region sequence. Evolution (N Y). 1997;51: 946–956. 10.1111/j.1558-5646.1997.tb03675.x 28568600

[28] Wright S. Isolation by distance. Genetics. 1943;28: 114–138. 17247074 10.1093/genetics/28.2.114 PMC1209196

[29] Jenkins D, Carey M, Czerniewska J, Fletcher J, Hether T, Jones A, et al A meta-analysis of isolation by distance: Relic or reference standard for landscape genetics? Ecography (Cop). 2010;33: 315–320.

[30] Hewitt GM. Genetic consequences of climatic oscillations in the Quaternary. Philos Trans R Soc B. 2004;359: 183–195. 10.1098/rstb.2003.1388 PMC1693318 15101575

[31] Carnaval AC, Hickerson MJ, Haddad CFB, Rodrigues MT, Moritz C. Stability predicts genetic diversity in the Brazilian Atlantic Forest Hotspot. Science. 2009;323: 785–789. 10.1126/science.1166955 19197066

[32] Ridgely RS TG. Vol 1, The Birds of South America. Austin: University of Texas Press; 1989.

[33] Alves MAS. Dieta e táticas de forrageamento de Neothraupis fasciata em cerrado no Distrito Federal, Brasil (Passeriformes: Emberizidae). Ararajuba. 1991;2: 25–29.

[34] Sick H. Ornitologia Brasileira (Edição rev.) Rio de Janeiro: Editora Nova Fronteira; 2001.

[35] Hilty S, de Juana E. White-banded Tanager (Neothraupis fasciata) In: del Hoyo J, Elliott A, Sargatal J, Christie DA, de Juana E, editors. Handbook of the Birds of the World Alive. Barcelona: Lynx Edicions; 2017.

[36] Alves MAS. Social system and helping behavior in the White-banded tanager (Neothraupis fasciata). Condor. 1990;92: 470–474.

[37] Duca C, Marini MA. Territorial system and adult dispersal in a cooperative-breeding tanager. Auk. 2014;131: 32–40.

[38] Manica LT, Marini MA. Helpers at the nest of White-banded Tanager Neothraupis fasciata benefit male breeders but do not increase reproductive success. J Ornithol. 2012;153: 149–159.

[39] Lima-Rezende CA, Souza RO, Caparroz R. The spatial genetic structure of the White-banded Tanager (Aves, Passeriformes) in fragmented Neotropical savannas suggests two evolutionarily significant units. Biotropica. 2019; doi:Forthcoming

[40] Kuhn M. Building Predictive Models in R Using the caret Package. J Stat Softw. 2008;28: 1–26. 10.18637/jss.v028.i07 27774042 PMC5074077

[41] R Core Team. R: A language and environment for statistical computing. Vienna, Austria: R Foundation for Statistical Computing; 2017.

[42] Phillips SJ, Anderson RP, Schapire RE. Maximum entropy modeling of species geographic distributions. Ecol Modell. 2006;190: 231–259.

[43] Hijmans RJ, Cameron SE, Parra JL, Jones PG, Jarvis A. Very high resolution interpolated climate surfaces for global land areas. Int J Climatol. 2005;25: 1965–1978.

[44] Otto-Bliesner BL, Marshall SJ, Overpeck JT, Miller GH, Hu A, CAPE LIP members. Simulating Arctic climate warmth and icefield retreat in the last interglaciation. Science. 2006;311: 1751–1753. 10.1126/science.1120808 16556838

[45] Danielson JJ, Gesch DB. Global multi-resolution terrain elevation data 2010 (GMTED2010) [Internet]. 2011. Available: https://pubs.usgs.gov/of/2011/1073/pdf/of2011-1073.pdf

[46] Conrad O, Bechtel B, Bock M, Dietrich H, Fischer E, Gerlitz L, et al System for Automated Geoscientific Analyses (SAGA) v. 2.1.4. Geosci Model Dev Discuss. 2015;8: 2271–2312.

[47] Forgy E. Cluster analysis of multivariate data: Efficiency vs. interpretability of classification. Biometrics. 1965;21: 768–769.

[48] Rubin J. Optimal classification into groups: An approach for solving the taxonomy problem. J Theor Biol. 1967;15: 103–144. 6034157 10.1016/0022-5193(67)90046-x

[49] Riitters K, Wickham J, O’neill R, Jones B, Smith E, Coulston J, et al Fragmentation of Continental United States Forests. Ecosystems. 2002;5: 815–822.

[50] Riitters K, Wickham J, O’neill R, Jones B, Smith E. Global-scale patterns of forest fragmentation. Conserv Ecol. 2000;4: 3.

[51] Birdlife International, NatureServe. Bird species distribution maps of the world. The IUCN Red List of Threatened Species Version 2015–3. [Internet]. 2014 [cited 14 Nov 2013]. Available: http://www.iucnredlist.org/

[52] Olson DM, Dinerstein E, Wikramanayake ED, Burgess ND, Powell GVN, Underwood EC, et al Terrestrial ecoregions of the world: A new map of life on earth. Bioscience. 2001;51: 933–938.

[53] Bruford MW, Hanotte O, Brookfield JFY, Burke T. Single-locus and multilocus DNA fingerprinting In: Hoelzel AR, editor. Molecular genetic analysis of populations. IRL Press; 1992 pp. 225–269.

[54] Ribas CC, Gaban-Lima R, Miyaki CY, Cracraft J. Historical biogeography and diversification within the Neotropical parrot genus Pionopsitta (Aves: Psittacidae). J Biogeogr. 2005;32: 1409–1427.

[55] Sorenson MD, Ast JC, Dimcheff DE, Yuri T, Mindell DP. Primers for a PCR-based approach to mitochondrial genome sequencing in birds and other vertebrates. Mol Phylogenet Evol. 1999;12: 105–14. 10.1006/mpev.1998.0602 10381314

[56] Marini M, Hackett S. A multifaceted approach to the characterization of an intergeneric hybrid manakin (Pipridae) from Brazil. Auk. 2002;119: 1114–1120.

[57] Dmitriev DA, Rakitov RA. Decoding of superimposed traces produced by direct sequencing of heterozygous indels. Plos Comput Biol. 2008;4: e1000113 10.1371/journal.pcbi.1000113 18654614 PMC2429969

[58] Librado P, Rozas J. DnaSP v5: A software for comprehensive analysis of DNA polymorphism data. Bioinformatics. 2009;25: 1451–1452. 10.1093/bioinformatics/btp187 19346325

[59] Bandelt HJ, Forster P, Röhl A. Median-joining networks for inferring intraspecific phylogenies. Mol Biol Evol. 1999;16: 37–48. 10.1093/oxfordjournals.molbev.a026036 10331250

[60] Leigh JW, Bryant D. PopART: full-feature software for haplotype network construction. Methods Ecol Evol. 2015;6: 1110–1116.

[61] Excoffier L, Lischer HEL. Arlequin suite ver 3.5: A new series of programs to perform population genetics analyses under Linux and Windows. Mol Ecol Resour. 2010;10: 564–567. 10.1111/j.1755-0998.2010.02847.x 21565059

[62] Posada D. Selection of models of DNA evolution with jModelTest. Methods Mol Biol. 2009;537: 93–112. 10.1007/978-1-59745-251-9_5 19378141

[63] Corander J, Sirén J, E A. Bayesian spatial modelling of genetic population structure. Comput Stat. 2008;23: 111–129.

[64] Jensen JL, Bohonak AJ, Kelley ST. Isolation by distance, web service. BMC Genet. 2005;6: 13 10.1186/1471-2156-6-13 15760479 PMC1079815

[65] Hammer Ø, Harper DAT, Ryan PD. PAST: Paleontological statistics software package for education and data analysis. Palaeontol Electron. 2001;4: 1–9.

[66] Tajima F. Statistical method for testing the neutral mutation hypothesis by DNA polymorphism. Genetics. 1989;123: 585–595. 2513255 10.1093/genetics/123.3.585 PMC1203831

[67] Fu YX. Statistical tests of neutrality of mutations against population growth, hitchhiking and background selection. Genetics. 1997;147: 915–925. 9335623 10.1093/genetics/147.2.915 PMC1208208

[68] Ramos-Onsins SE, Rozas J. Statistical properties of new neutrality tests against population growth. Mol Biol Evol. 2002;19: 2092–2100. 10.1093/oxfordjournals.molbev.a004034 12446801

[69] Drummond AJ, Suchard MA, Xie D, Rambaut A. Bayesian phylogenetic with BEAUti and the BEAST 1.7. Mol Biol Evol. 2012;29: 1969–1973. 10.1093/molbev/mss075 22367748 PMC3408070

[70] Pacheco MA, Battistuzzi FU, Lentino M, Aguilar RF, Kumar S, Scalante AA. Evolution of modern birds revealed by mitogenomics: Timing the radiation and origin of major orders. Mol Biol Evol. 2011;28: 1927–1942. 10.1093/molbev/msr014 21242529 PMC3144022

[71] Axelsson E, Smith NGC, Sundstrom H, Berlin S, Ellegren H. Male-biased mutation rate and divergence in autosomal, Z-linked and W-linked introns of chicken and turkey. Mol Biol Evol. 2004;21: 1538–1547. 10.1093/molbev/msh157 15140948

[72] Rambaut A, Suchard M, Drummond AJ. Tracer. 2013.

[73] King LA. Geomorfologia do Brasil Oriental. Rev Bras Geogr. 1956;18: 147–265.

[74] Braun OPG. Contribuição à geomorfologia do Brasil Central. Rev Bras Geogr. 1970;32: 3–39.

[75] King LC. A geomorphological comparison between Eastern Brazil and Africa (Central and Southern). Quat J Geol Soc. 1956;112: 445–474.

[76] Cheviron ZA, Hackett SJ, Capparella AP. Complex evolutionary history of a Neotropical lowland forest bird (Lepidothrix coronata) and its implications for historical hypotheses of the origin of Neotropical avian diversity. Mol Phylogenet Evol. 2005;36: 338–357. 10.1016/j.ympev.2005.01.015 15955514

[77] Burns KJ, Shultz AJ, Title PO, Mason NA, Barker FK, Klicka J, et al Phylogenetics and diversification of tanagers (Passeriformes: Thraupidae), the largest radiation of Neotropical songbirds. Mol Phylogenet Evol. 2014;75: 41–77. 10.1016/j.ympev.2014.02.006 24583021

[78] Hilty S, Bonan A. Tanagers (Thraupidae) In: del Hoyo J, Elliott A, Sartagal J, Christie DA, de Juana E, editors. Handbook of the Birds of the World Alive. Barcelona: Lynx Edicions; 2017.

[79] Crispo E, Hendry A. Does time since colonization influence isolation by distance? A meta-analysis. Conserv Genet. 2005;6: 665–682.

[80] Castric V, Bernatchez L. The rise and fall of isolation by distance in the anadromous brook charr (Salvelinus fontinalis Mitchill). Genetics. 2003;163: 983–996. 12663537 10.1093/genetics/163.3.983 PMC1462472

[81] Ab’Sáber A. O domínio dos cerrados: Introdução ao conhecimento. Rev do Serviço Público. 1983;111: 41–55.

[82] Santos MG, Nogueira C, Giugliano LG, Colli GR. Landscape evolution and phylogeography of Micrablepharus atticolus (Squamata, Gymnophthalmidae), an endemic lizard of the Brazilian Cerrado. J Biogeogr. 2014;41: 1506–1519.

[83] Buzatti RS de O, Lemos-Filho JP, Bueno ML, Lovato MB. Multiple Pleistocene refugia in the Brazilian cerrado: evidence from phylogeography and climatic nichemodelling of two Qualea species (Vochysiaceae). Bot J Linn Soc. 2017;185: 307–320.

[84] Haffer J, Prance GT. Impulsos climáticos da evolução na Amazônia durante o Cenozóico: Sobre a teoria dos Refúgios da diferenciação biótica. Estud Avançados. 2002;16: 175–206.

[85] Van Andel TH. New views on an old planet: A history of global change London: Cambridge University Press; 1985.

[86] K M Cohen, Gibbard PL. Global chronostratigraphical correlation table for the last 2.7 million years. v. 2016a [Internet]. 2016 [cited 24 Oct 2018]. Available: http://www.stratigraphy.org/upload/QuaternaryChart1.JPG

