# Supplementary material for: Late Pleistocene climatic changes promoted demographic expansion and population reconnection of a Neotropical savanna-adapted bird, Neothraupis fasciata (Aves: Thraupidae)
Source: PLoS One. 2019 Mar 20;14(3):e0212876. doi: 10.1371/journal.pone.0212876 (PMC6426193; doi:10.1371/journal.pone.0212876)
Supplement: S1 Table — (DOCX) [file pone.0212876.s001.docx]

**Supporting Information**

**S1 Table. *Neothraupis fasciata* occurrence points.**

| **State (Country)** | **Latitude** | **Longitude** |
| --- | --- | --- |
| Amambay (Paraguay) | -23 | -56 |
| Amapá (Brazil) | 0.4833 | -51.1 |
| Amapá (Brazil) | 0.0333 | -51.05 |
| Bahia (Brazil) | -13.75 | -45.6 |
| Bahia (Brazil) | -13.1 | -41.4833 |
| Bahia (Brazil) | -13.04 | -41.42 |
| Bahia (Brazil) | -11.40861111 | -41.16477778 |
| Bahia (Brazil) | -10.50536111 | -41.33102778 |
| Bahia (Brazil) | -9.691944444 | -40.55763889 |
| Caazapa (Paraguay) | -25.4333 | -56.0333 |
| Caazapa (Paraguay) | -25.0333 | -55.85 |
| Concepcion (Bolivia) | -16.1131113 | -61.98349 |
| Distrito Federal (Brazil) | -15.7833 | -47.9167 |
| Distrito Federal (Brazil) | -15.95 | -47.9167 |
| Distrito Federal (Brazil) | -15.7167 | -47.95 |
| Distrito Federal (Brazil) | -15.6167 | -47.6667 |
| Distrito Federal (Brazil) | -15.941 | -47.866 |
| Distrito Federal (Brazil) | -15.5574 | -47.605 |
| Divisa Maranhão-Tocantins (Brazil) | -9.469611111 | -46.59766667 |
| Goiás (Brazil) | -16.3333 | -48.9667 |
| Goiás (Brazil) | -15.9167 | -52.25 |
| Goiás (Brazil) | -14.1167 | -47.5167 |
| Goiás (Brazil) | -16.2667 | -42.9167 |
| Goiás (Brazil) | -16.25 | -52.4167 |
| Goiás (Brazil) | -15.5333 | -49.45 |
| Goiás (Brazil) | -17.8833 | -51.7167 |
| Goiás (Brazil) | -13.6167 | -48.9 |
| Goiás (Brazil) | -16.0167 | -49.7833 |
| Goiás (Brazil) | -16.4167 | -49.2333 |
| Goiás (Brazil) | -17.7833 | -48.7 |
| Goiás (Brazil) | -18.2609794 | -52.8883982 |
| Goiás (Brazil) | -14.1833 | -47.8 |
| Goiás (Brazil) | -14.7 | -47.5333 |
| Goiás (Brazil) | -18.16 | -52.93 |
| Maranhão (Brazil) | -6.1167 | -45.15 |
| Maranhão (Brazil) | -9.1 | -45.95 |
| Maranhão (Brazil) | -5.5 | -45.25 |
| Maranhão (Brazil) | -9.5833 | -46.4167 |
| Maranhão (Brazil) | -8.75 | -45.9667 |
| Maranhão (Brazil) | -6.4167 | -45.1667 |
| Maranhão (Brazil) | -7.7667 | -46.2333 |
| Maranhão (Brazil) | -7.21 | -47.47 |
| Mato Grosso (Brazil) | -15.318625 | -56.0055542 |
| Mato Grosso (Brazil) | -15.3 | -55.8333 |
| Mato Grosso (Brazil) | -15.4667 | -55.55 |
| Mato Grosso (Brazil) | -15.6412 | -56.612 |
| Mato Grosso (Brazil) | -15.4536 | -55.7666 |
| Mato Grosso (Brazil) | -12.8333 | -58.4667 |
| Mato Grosso (Brazil) | -13.3833 | -51.3667 |
| Mato Grosso (Brazil) | -17.2 | -54.17 |
| Mato Grosso (Brazil) | -14.61 | -52.27 |
| Mato Grosso (Brazil) | -15.4 | -55.82 |
| Mato Grosso do Sul (Brazil) | -20.45 | -54.6167 |
| Mato Grosso do Sul (Brazil) | -18.1 | -53.3 |
| Mato Grosso do Sul (Brazil) | -18.4667 | -57.3667 |
| Mato Grosso do Sul (Brazil) | -15.65 | -57.2167 |
| Mato Grosso do Sul (Brazil) | -20.7833 | -51.7 |
| Mato Grosso do Sul (Brazil) | -19.505 | -55.61222222 |
| Minas Gerais (Brazil) | -18.8833 | -47.6333 |
| Minas Gerais (Brazil) | -20.0167 | -43.9833 |
| Minas Gerais (Brazil) | -16.7333 | -42.9667 |
| Minas Gerais (Brazil) | -16.8667 | -43.0167 |
| Minas Gerais (Brazil) | -16.45 | -43.4 |
| Minas Gerais (Brazil) | -18.25 | -43.6 |
| Minas Gerais (Brazil) | -19.3667 | -43.7 |
| Minas Gerais (Brazil) | -17.0667 | -45.9 |
| Minas Gerais (Brazil) | -17.1167 | -42.7667 |
| Minas Gerais (Brazil) | -18.1833 | -45.4167 |
| Minas Gerais (Brazil) | -15.8333 | -46.5 |
| Minas Gerais (Brazil) | -14.9333 | -46.2167 |
| Minas Gerais (Brazil) | -16.5667 | -42.9 |
| Minas Gerais (Brazil) | -15.4167 | -44.4333 |
| Minas Gerais (Brazil) | -19.6333 | -43.8833 |
| Minas Gerais (Brazil) | -15.9333 | -44.8667 |
| Minas Gerais (Brazil) | -19.9333 | -43.8833 |
| Minas Gerais (Brazil) | -15.35 | -45.8333 |
| Minas Gerais (Brazil) | -20.3338333 | -46.775 |
| Minas Gerais (Brazil) | -20.2506019 | -46.4955139 |
| Minas Gerais (Brazil) | -15.6667 | -44.6333 |
| Minas Gerais (Brazil) | -18.95 | -48.2 |
| Minas Gerais (Brazil) | -20.1 | -43.9833 |
| Minas Gerais (Brazil) | -19.35 | -43.6167 |
| Minas Gerais (Brazil) | -16.6833 | -43.3167 |
| Minas Gerais (Brazil) | -19.5833 | -43.9 |
| Minas Gerais (Brazil) | -16.8667 | -42.9833 |
| Minas Gerais (Brazil) | -19.1659243 | -48.581543 |
| Minas Gerais (Brazil) | -16.6167 | -43.3 |
| Minas Gerais (Brazil) | -16.3667 | -45.0667 |
| Minas Gerais (Brazil) | -20.25 | -46.6167 |
| Minas Gerais (Brazil) | -16.5 | -42.9 |
| Minas Gerais (Brazil) | -16.55 | -43.4 |
| Minas Gerais (Brazil) | -16.6667 | -49.5 |
| Minas Gerais (Brazil) | -19.1833 | -48.35 |
| Minas Gerais (Brazil) | -17.64111111 | -43.50083333 |
| Minas Gerais (Brazil) | -9.900277778 | -41.51 |
| Minas Gerais (Brazil) | -20.14 | -46.71 |
| Minas Gerais (Brazil) | -16.84 | -43.53 |
| Minas Gerais (Brazil) | -17.028 | -45.7 |
| Minas Gerais (Brazil) | -15.094573 | -45.530577 |
| Pará (Brazil) | -9.666666667 | -50.38333333 |
| Paraná (Brazil) | -24.25 | -49.8 |
| Paraná (Brazil) | -24.5 | -50.33333333 |
| Piauí (Brazil) | -8.2 | -45.6167 |
| Piauí (Brazil) | -6.7833 | -43.8333 |
| Piauí (Brazil) | -8.8667 | -44.9667 |
| Piauí (Brazil) | -8.7 | -45.0167 |
| Piauí (Brazil) | -10 | -45.8 |
| Piauí (Brazil) | -9.6333 | -45.45 |
| Piauí (Brazil) | -9.8333 | -45.35 |
| Piauí (Brazil) | -7.236 | -44.43 |
| San Pedro (Paraguay) | -23.8167 | -56.3 |
| Santa Cruz (Bolivia) | -18 | -60 |
| Santa Cruz (Bolivia) | -16.25 | -62.0667 |
| Santa Cruz (Bolivia) | -13.9667 | -60.8333 |
| Santa Cruz (Bolivia) | -14.525 | -60.7398 |
| Santa Cruz (Bolivia) | -14.5167 | -60.3833 |
| Santa Cruz (Bolivia) | -18.0333 | -60.1833 |
| São Paulo (Brazil) | -22.85 | -48.9333 |
| São Paulo (Brazil) | -23.4 | -48.3667 |
| São Paulo (Brazil) | -22.3167 | -49.0667 |
| São Paulo (Brazil) | -22.1667 | -47.8667 |
| São Paulo (Brazil) | -21.8167 | -48.2 |
| São Paulo (Brazil) | -23.5667 | -47.9 |
| São Paulo (Brazil) | -21.9167 | -47.8167 |
| São Paulo (Brazil) | -21.2167 | -47.2833 |
| São Paulo (Brazil) | -22.1 | -47.85 |
| São Paulo (Brazil) | -22.8 | -49.2167 |
| São Paulo (Brazil) | -22.2667 | -47.1667 |
| São Paulo (Brazil) | -23.6333 | -47.9667 |
| São Paulo (Brazil) | -20.85 | -47.5833 |
| São Paulo (Brazil) | -22.8833 | -49.25 |
| São Paulo (Brazil) | -22.9667 | -48.8667 |
| São Paulo (Brazil) | -22.1667 | -47.9167 |
| São Paulo (Brazil) | -23.1167 | -48.9833 |
| São Paulo (Brazil) | -20.5333 | -47.4 |
| São Paulo (Brazil) | -23.7167 | -47.9833 |
| São Paulo (Brazil) | -24.1167 | -49.3333 |
| São Paulo (Brazil) | -20.8 | -47.7333 |
| São Paulo (Brazil) | -22.3 | -47.85 |
| São Paulo (Brazil) | -22.2833 | -47.8 |
| São Paulo (Brazil) | -22.2 | -46.95 |
| São Paulo (Brazil) | -23.0833 | -48.3833 |
| São Paulo (Brazil) | -22.3 | -48.0167 |
| São Paulo (Brazil) | -21.5833 | -48.0667 |
| São Paulo (Brazil) | -22.25 | -47.8667 |
| São Paulo (Brazil) | -20.1167 | -47.8333 |
| São Paulo (Brazil) | -23.53472222 | -47.51619444 |
| São Paulo (Brazil) | -22.45 | -48.95 |
| Tocantins (Brazil) | -5.6333 | -48.1167 |
| Tocantins (Brazil) | -11.3 | -48.9333 |
| Tocantins (Brazil) | -10.4667 | -46.3167 |
| Tocantins (Brazil) | -12.4 | -47.6833 |
| Tocantins (Brazil) | -10.2667 | -48.3667 |
| Tocantins (Brazil) | -9.55 | -48.3667 |
| Tocantins (Brazil) | -13.39602778 | -47.70555556 |
| Tocantins (Brazil) | -8.95 | -47.33333333 |
| Tocantins (Brazil) | -10.76747222 | -47.48619444 |
| Sipaliwini (Suriname) | 1.9633 | -55.9282 |
