# Supplementary material for: Late Pleistocene climatic changes promoted demographic expansion and population reconnection of a Neotropical savanna-adapted bird, Neothraupis fasciata (Aves: Thraupidae)
Source: PLoS One. 2019 Mar 20;14(3):e0212876. doi: 10.1371/journal.pone.0212876 (PMC6426193; doi:10.1371/journal.pone.0212876)
Supplement: S2 Table — The average Area Under the ROC curve (AUC) for the replicate runs (standard deviations in brackets) is given for each model. The average test omission rate (OR) is also given for each model. (DOCX) [file pone.0212876.s002.docx]

**Supporting Information**

**S2 Table. Summary of performance statistics of *Neothraupis fasciata* MAXENT models.** The average Area Under the ROC curve (AUC) for the replicate runs (standard deviations in brackets) is given for each model. The average test omission rate (OR) is also given for each model.

| **Model** | **Test AUC (±SD)** | **Test OR** | **Test OR thresholded** |
| --- | --- | --- | --- |
| Present | 0.9054 ± 0.0211 | 0.0178 | 0.1919 |
| Holocene - MIROC | 0.918 ± 0.019 | 0.0113 | 0.1742 |
| Holocene - CCSM4 | 0.9134 ± 0.0202 | 0.0177 | 0.1952 |
| LGM - MIROC | 0.9073 ± 0.0216 | 0.0161 | 0.2064 |
| LGM - CCSM4 | 0.9006 ± 0.0228 | 0.0226 | 0.2016 |
| LIG | 0.9042 ± 0.022 | 0.0323 | 0.2242 |

MIROC and CCSM4 are General Circulation Models.

LGM – Last Glacial Maximum

LIG – Last Interglacial
