# Supplementary material for: Late Pleistocene climatic changes promoted demographic expansion and population reconnection of a Neotropical savanna-adapted bird, Neothraupis fasciata (Aves: Thraupidae)
Source: PLoS One. 2019 Mar 20;14(3):e0212876. doi: 10.1371/journal.pone.0212876 (PMC6426193; doi:10.1371/journal.pone.0212876)
Supplement: S3 Table — See Fig 4 for haplotype number (Hap) interpretation. Área de Proteção Ambiental das Bacias do Gama e Cabeça de Veado (AGCV); Brasilândia de Minas (BRMI); Parque Nacional da Chapada das Mesas (PNCM); Estação Ecológica de Águas Emendadas (EEAE); Embrapa Macapá Experimental Farm (MACA); Ouro e Prata Farm at Nova Xavantina (NXAV); Nova Aliança Farm at Ponte Alta do Tocantins (PATO); Parque Nacional Grande Sertão Veredas (PGSV); Parque Nacional Chapada dos Guimarães (PNCG); Parque Nacional das Emas (PNEM); Parque Nacional Serra da Canastra (PNSC); and Uruçuí (URUC). (DOCX) [file pone.0212876.s003.docx]

**Supporting Information**

**S3 Table. Genbank accession numbers and haplotypes for NADH dehydrogenase subunit 2 gene (ND2) and beta-fibrinogen gene intron 5 (FIB-5) datasets from *Neothraupis fasciata* individuals sampled along 12 different sample sites**. See Fig 4 for haplotype number (Hap) interpretation. Área de Proteção Ambiental das Bacias do Gama e Cabeça de Veado (AGCV); Brasilândia de Minas (BRMI); Parque Nacional da Chapada das Mesas (PNCM); Estação Ecológica de Águas Emendadas (EEAE); Embrapa Macapá Experimental Farm (MACA); Ouro e Prata Farm at Nova Xavantina (NXAV); Nova Aliança Farm at Ponte Alta do Tocantins (PATO); Parque Nacional Grande Sertão Veredas (PGSV); Parque Nacional Chapada dos Guimarães (PNCG); Parque Nacional das Emas (PNEM); Parque Nacional Serra da Canastra (PNSC); and Uruçuí (URUC).

| **ND2 Accession (Hap)** | **FIB-5 Accession (Hap)** | **Sample sites** |
| --- | --- | --- |
| MH277700 (Hap_1) | MH277805 (Hap_1; Hap_2) | AGCV |
| MH277701 (Hap_2) | MH277806 (Hap_2) | AGCV |
| MH277702 (Hap_2) | MH277807 (Hap_2; Hap_3) | AGCV |
| MH277703 (Hap_3) | MH277808 (Hap_2; Hap_3) | AGCV |
| MH277704 (Hap_2) | MH277809 (Hap_1; Hap_2) | AGCV |
| MH277705 (Hap_2) | MH277810 (Hap_2; Hap_3) | AGCV |
| MH277706 (Hap_4) | MH277811 (Hap_1; Hap_2) | AGCV |
| MH277707 (Hap_4) | MH277812 (Hap_2; Hap_3) | AGCV |
| MH277708 (Hap_5) | MH277813 (Hap_2; Hap_3) | AGCV |
| MH277709 (Hap_6) | MH277814 (Hap_3; Hap_4) | AGCV |
| MH277710 (Hap_3) | MH277815 (Hap_3; Hap_5) | AGCV |
| MH277711 (Hap_7) | MH277816 (Hap_1; Hap_2) | AGCV |
| MH277712 (Hap_8) | MH277817 (Hap_1; Hap_2) | AGCV |
| MH277713 (Hap_9) | MH277818 (Hap_1; Hap_3) | BRMI |
| MH277714 (Hap_10) | MH277819 (Hap_6; Hap_7) | BRMI |
| MH277715 (Hap_9) | MH277820 (Hap_1; Hap_3) | BRMI |
| MH277716 (Hap_8) | MH277821 (Hap_1; Hap_2) | PNCM |
| MH277717 (Hap_11) | MH277822 (Hap_2) | PNCM |
| MH277718 (Hap_8) | MH277823 (Hap_3) | PNCM |
| MH277719 (Hap_8) | MH277824 (Hap_3) | PNCM |
| MH277720 (Hap_8) | MH277825 (Hap_2; Hap_3) | PNCM |
| MH277721 (Hap_12) | MH277826 (Hap_2; Hap_3) | EEAE |
| MH277722 (Hap_3) | MH277827 (Hap_2; Hap_3) | EEAE |
| MH277723 (Hap_8) | MH277828 (Hap_1; Hap_3) | EEAE |
| MH277724 (Hap_2) | MH277829 (Hap_1; Hap_2) | EEAE |
| MH277725 (Hap_13) | MH277830 (Hap_1; Hap_2) | EEAE |
| MH277726 (Hap_8) | MH277831 (Hap_1; Hap_8) | EEAE |
| MH277727 (Hap_14) | MH277832 (Hap_4) | EEAE |
| MH277728 (Hap_15) | MH277833 (Hap_2; Hap_9) | MACA |
| MH277729 (Hap_8) | MH277834 (Hap_3) | MACA |
| MH277730 (Hap_15) | MH277835 (Hap_2; Hap_3) | MACA |
| MH277731 (Hap_15) | MH277836 (Hap_3) | MACA |
| MH277732 (Hap_8) | MH277837 (Hap_2; Hap_3) | MACA |
| MH277733 (Hap_15) | MH277838 (Hap_2; Hap_9) | MACA |
| MH277734 (Hap_15) | MH277839 (Hap_2) | MACA |
| MH277735 (Hap_15) | MH277840 (Hap_2; Hap_10) | MACA |
| MH277736 (Hap_8) | MH277841 (Hap_1; Hap_2) | MACA |
| MH277737 (Hap_16) | MH277842 (Hap_1; Hap_3) | NXAV |
| MH277738 (Hap_8) | MH277843 (Hap_2; Hap_3) | NXAV |
| MH277739 (Hap_8) | MH277844 (Hap_2; Hap_11) | PATO |
| MH277740 (Hap_8) | MH277845 (Hap_1; Hap_3) | PATO |
| MH277741 (Hap_15) | MH277846 (Hap_1; Hap_3) | PATO |
| MH277742 (Hap_15) | MH277847 (Hap_3) | PATO |
| MH277743 (Hap_17) | MH277848 (Hap_1; Hap_3) | PATO |
| MH277744 (Hap_18) | MH277849 (Hap_1; Hap_12) | PATO |
| MH277745 (Hap_15) | MH277850 (Hap_4; Hap_13) | PATO |
| MH277746 (Hap_8) | MH277851 (Hap_3; Hap_4) | PATO |
| MH277747 (Hap_19) | MH277852 (Hap_3; Hap_14) | PATO |
| MH277748 (Hap_20) | MH277853 (Hap_3 | PATO |
| MH277749 (Hap_21) | MH277854 (Hap_15; Hap_16) | PATO |
| MH277750 (Hap_20) | MH277855 (Hap_3) | PATO |
| MH277751 (Hap_22) | MH277856 (Hap_1; Hap_12) | PATO |
| MH277752 (Hap_8) | MH277857 (Hap_1; Hap_17) | PATO |
| MH277753 (Hap_9) | MH277858 (Hap_1; Hap_3) | PGSV |
| MH277754 (Hap_2) | MH277859 (Hap_1; Hap_3) | PGSV |
| MH277755 (Hap_9) | MH277860 (Hap_1; Hap_3) | PGSV |
| MH277756 (Hap_2) | MH277861 (Hap_3; Hap_9) | PGSV |
| MH277757 (Hap_8) | MH277862 (Hap_11) | PGSV |
| MH277758 (Hap_23) | MH277863 (Hap_9; Hap_12) | PGSV |
| MH277759 (Hap_8) | MH277864 (Hap_1; Hap_2) | PGSV |
| MH277760 (Hap_8) | MH277865 (Hap_3) | PGSV |
| MH277761 (Hap_2) | MH277866 (Hap_2; Hap_3) | PGSV |
| MH277762 (Hap_24) | MH277867 (Hap_12) | PGSV |
| MH277763 (Hap_25) | MH277868 (Hap_12) | PGSV |
| MH277764 (Hap_25) | MH277869 (Hap_2; Hap_3) | PGSV |
| MH277765 (Hap_26) | MH277870 (Hap_1; Hap_2) | PGSV |
| MH277766 (Hap_8) | MH277871 (Hap_2; Hap_3) | PGSV |
| MH277767 (Hap_9) | MH277872 (Hap_2) | PGSV |
| MH277768 (Hap_9) | MH277873 (Hap_2; Hap_3) | PGSV |
| MH277769 (Hap_9) | MH277874 (Hap_12; Hap_18) | PGSV |
| MH277770 (Hap_8) | MH277875 (Hap_2; Hap_12) | PGSV |
| MH277771 (Hap_2) | MH277876 (Hap_1; Hap_12) | PGSV |
| MH277772 (Hap_9) | MH277877 (Hap_1; Hap_3) | PGSV |
| MH277773 (Hap_27) | MH277878 (Hap_1; Hap_3) | PGSV |
| MH277774 (Hap_8) | MH277879 (Hap_2; Hap_3) | PGSV |
| MH277775 (Hap_28) | MH277880 (Hap_2; Hap_3) | PNCG |
| MH277776 (Hap_2) | MH277881 (Hap_1; Hap_3) | PNCG |
| MH277777 (Hap_29) | MH277882 (Hap_3; Hap_8) | PNCG |
| MH277778 (Hap_30) | MH277883 (Hap_3) | PNCG |
| MH277779 (Hap_29) | MH277884 (Hap_3) | PNCG |
| MH277780 (Hap_29) | MH277885 (Hap_3) | PNCG |
| MH277781 (Hap_8) | MH277886 (Hap_1; Hap_3) | PNCG |
| MH277782 (Hap_31) | MH277887 (Hap_1; Hap_2) | PNCG |
| MH277783 (Hap_32) | MH277888 (Hap_3) | PNEM |
| MH277784 (Hap_33) | MH277889 (Hap_3) | PNEM |
| MH277785 (Hap_34) | MH277890 (Hap_3; Hap_12) | PNEM |
| MH277786 (Hap_8) | MH277891 (Hap_2; Hap_3) | PNEM |
| MH277787 (Hap_8) | MH277892 (Hap_2; Hap_3) | PNEM |
| MH277788 (Hap_8) | MH277893 (Hap_19; Hap_20) | PNEM |
| MH277789 (Hap_8) | MH277894 (Hap_2; Hap_3) | PNEM |
| MH277790 (Hap_8) | MH277895 (Hap_21; Hap_22) | PNEM |
| MH277791 (Hap_8) | MH277896 (Hap_1; Hap_3) | PNEM |
| MH277792 (Hap_8) | MH277897 (Hap_2; Hap_3) | PNSC |
| MH277793 (Hap_8) | MH277898 (Hap_2; Hap_12) | PNSC |
| MH277794 (Hap_35) | MH277899 (Hap_1; Hap_3) | PNSC |
| MH277795 (Hap_8) | MH277900 (Hap_2) | PNSC |
| MH277796 (Hap_8) | MH277901 (Hap_3) | PNSC |
| MH277797 (Hap_36) | MH277902 (Hap_2; Hap_3) | PNSC |
| MH277798 (Hap_36) | MH277903 (Hap_2; Hap_3) | PNSC |
| MH277799 (Hap_35) | MH277904 (Hap_2; Hap_3) | PNSC |
| MH277800 (Hap_36) | MH277905 (Hap_12; Hap_23) | PNSC |
| MH277801 (Hap_8) | MH277906 (Hap_15; Hap_17) | URUC |
| MH277802 (Hap_8) | MH277907 (Hap_1; Hap_2) | URUC |
| MH277803 (Hap_17) | MH277908 (Hap_1; Hap_2) | URUC |
| MH277804 (Hap_8) | MH277909 (Hap_1; Hap_3) | URUC |
