# Supplementary material for: Late Pleistocene climatic changes promoted demographic expansion and population reconnection of a Neotropical savanna-adapted bird, Neothraupis fasciata (Aves: Thraupidae)
Source: PLoS One. 2019 Mar 20;14(3):e0212876. doi: 10.1371/journal.pone.0212876 (PMC6426193; doi:10.1371/journal.pone.0212876)
Supplement: S4 Table — Significant p-values after False Discovery Rate correction are marked with one asterisk (*). (DOCX) [file pone.0212876.s004.docx]

**Supporting Information**

**S4 Table. Pairwise *F*_ST_ values for NADH dehydrogenase subunit 2 gene (bellow diagonal) and beta-fibrinogen gene intron 5 (above diagonal) datasets for *Neothraupis fasciata* sample sites (see Fig 1 for abbreviations).** Significant p-values after False Discovery Rate correction are marked with asterisk (*).

|  | AGCV | BRMI | EEAE | MACA | NXAV | PATO | PGSV | PNCG | PNCM | PNEM | PNSC | URUC |
| --- | --- | --- | --- | --- | --- | --- | --- | --- | --- | --- | --- | --- |
| AGCV |  | 0.227 | -0.039 | -0.003 | -0.037 | 0.056 | 0.084 | 0.148 | -0.008 | 0.091 | 0.033 | 0.023 |
| BRMI | 0.597 |  | 0.174 | 0.205 | -0.052 | -0.014 | 0.017 | 0.140 | 0.097 | -0.009 | 0.025 | -0.033 |
| EEAE | 0.011 | 0.566 |  | 0.014 | -0.029 | 0.041 | 0.069 | 0.160 | 0.011 | 0.085 | 0.025 | 0.001 |
| MACA | 0.274* | 0.750 | 0.295 |  | -0.034 | 0.056 | 0.074 | 0.130 | -0.034 | 0.054 | 0.012 | 0.054 |
| NXAV | 0.120 | 0.399 | 0.083 | 0.552 |  | -0.096 | -0.003 | -0.153 | -0.183 | -0.115 | -0.057 | -0.095 |
| PATO | 0.134* | 0.665 | 0.083 | 0.112 | 0.158 |  | 0.004 | 0.042 | -0.007 | -0.019 | -0.018 | -0.060 |
| PGSV | 0.342* | -0.091 | 0.299 | 0.364 | 0.216 | 0.349* |  | 0.134 | 0.054 | 0.027 | -0.024 | -0.045 |
| PNCG | 0.112 | 0.555 | 0.072 | 0.306 | 0.027 | 0.085 | 0.246 |  | -0.017 | 0.009 | 0.108 | 0.109 |
| PNCM | 0.090 | 0.573 | 0.033 | 0.352 | 0.119 | 0.026 | 0.273 | 0.045 |  | -0.023 | -0.002 | 0.001 |
| PNEM | 0.105 | 0.534 | 0.035 | 0.208 | 0.026 | 0.035 | 0.273 | -0.005 | -0.043 |  | -0.005 | -0.021 |
| PNSC | 0.183* | 0.410 | 0.097 | 0.250 | 0.020 | 0.160 | 0.268 | 0.077 | 0.050 | 0.005 |  | -0.068 |
| URUC | -0.003 | 0.593 | -0.010 | 0.455 | 0.258 | -0.070 | 0.248 | 0.000 | -0.029 | -0.086 | 0.031 |  |
