# Supplementary material for: Late Pleistocene climatic changes promoted demographic expansion and population reconnection of a Neotropical savanna-adapted bird, Neothraupis fasciata (Aves: Thraupidae)
Source: PLoS One. 2019 Mar 20;14(3):e0212876. doi: 10.1371/journal.pone.0212876 (PMC6426193; doi:10.1371/journal.pone.0212876)
Supplement: S1 Fig — Warmer colors represent areas of higher habitat suitability. (DOCX) [file pone.0212876.s005.docx]

**Supporting Information**


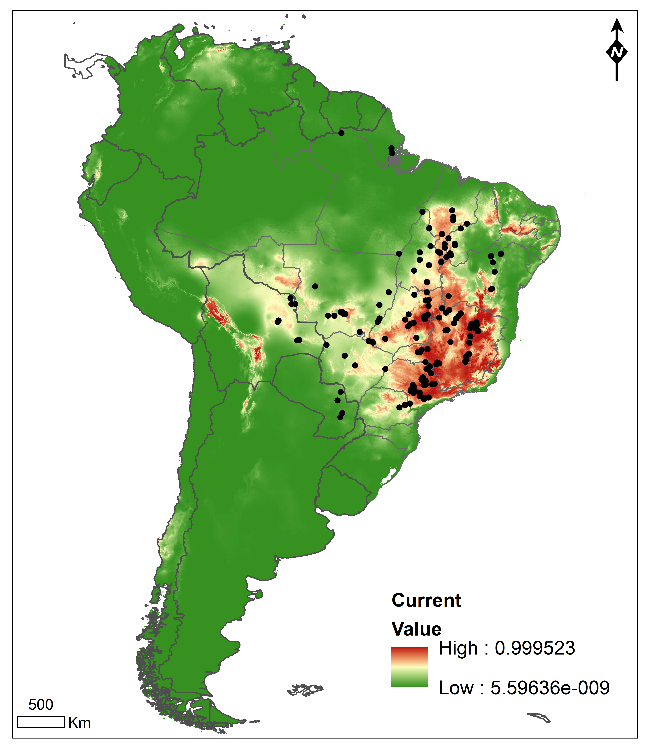


**S1 Fig. *Neothraupis fasciata* occurrence points and predicted present** **distribution based on the set of seven non-collinear variables (Bio2, Bio3, Bio10, Bio13, Bio15, Bio18, and Bio19).** Warmer colors represent areas of higher habitat suitability.
