# Supplementary material for: Late Pleistocene climatic changes promoted demographic expansion and population reconnection of a Neotropical savanna-adapted bird, Neothraupis fasciata (Aves: Thraupidae)
Source: PLoS One. 2019 Mar 20;14(3):e0212876. doi: 10.1371/journal.pone.0212876 (PMC6426193; doi:10.1371/journal.pone.0212876)
Supplement: S2 Fig — Neothraupis fasciata occurrence points and predicted Holocene distribution using (A) MIROC and (B) CCSM4 General Circulation Models. Warmer colors represent areas of higher habitat suitability. (DOCX) [file pone.0212876.s006.docx]

**Supporting Information**


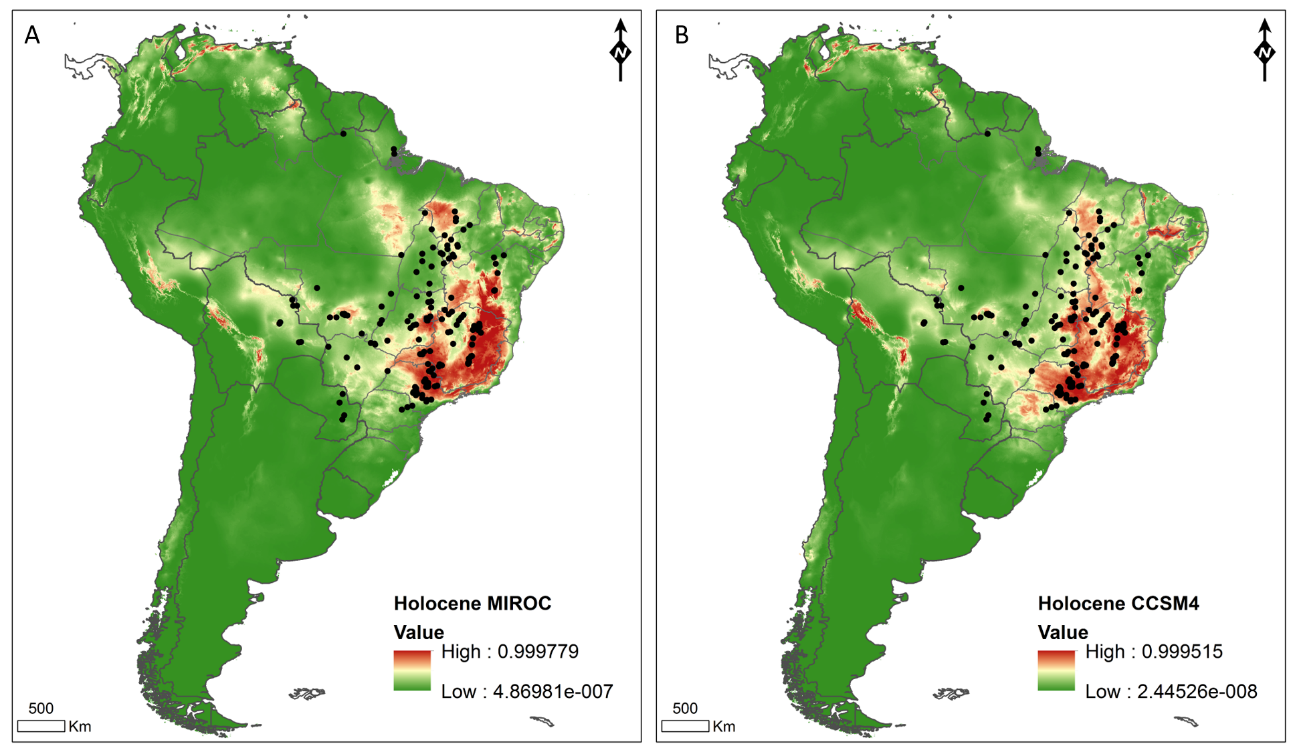


**S2 Fig. *Neothraupis fasciata* occurrence records and predicted Holocene distribution using (A) MIROC and (B) CCSM4 General Circulation Models.** Warmer colors represent areas of higher habitat suitability.
