# Supplementary material for: Late Pleistocene climatic changes promoted demographic expansion and population reconnection of a Neotropical savanna-adapted bird, Neothraupis fasciata (Aves: Thraupidae)
Source: PLoS One. 2019 Mar 20;14(3):e0212876. doi: 10.1371/journal.pone.0212876 (PMC6426193; doi:10.1371/journal.pone.0212876)
Supplement: S4 Fig — Warmer colors represent areas of higher habitat suitability. (DOCX) [file pone.0212876.s008.docx]

**Supporting Information**


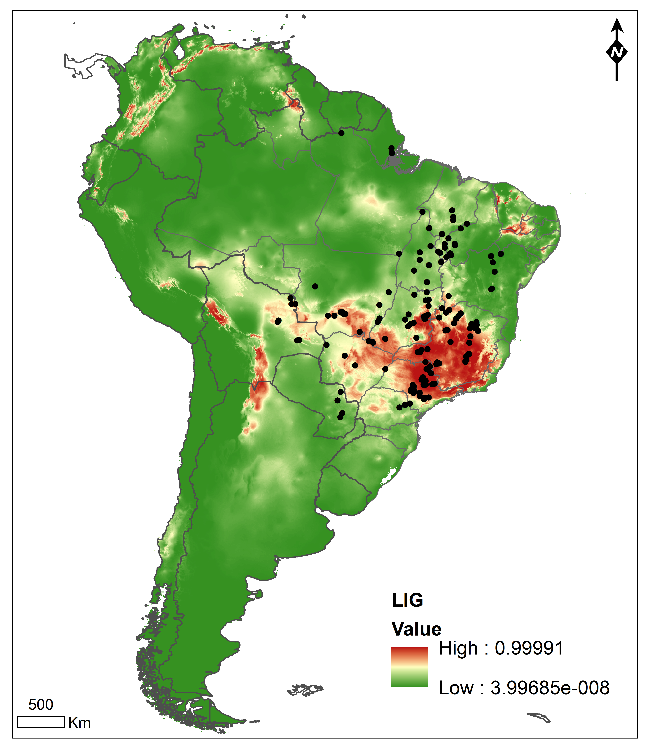


**S4 Fig.** ***Neothraupis fasciata* occurrence points and predicted Last Interglacial distribution.** Warmer colors represent areas of higher habitat suitability.
